# Regulation of Histone Acetylation Modification on Biosynthesis of Secondary Metabolites in Fungi

**DOI:** 10.3390/ijms26010025

**Published:** 2024-12-24

**Authors:** Xuwen Hou, Liyao Liu, Yu Li, Pengfei Wang, Xiaoqian Pan, Dan Xu, Daowan Lai, Ligang Zhou

**Affiliations:** Department of Plant Pathology, College of Plant Protection, China Agricultural University, Beijing 100193, China; xwhou@cau.edu.cn (X.H.); lyliu@cau.edu.cn (L.L.); yuli@cau.edu.cn (Y.L.); pengfeiwang@cau.edu.cn (P.W.); xiaoqianpan@cau.edu.cn (X.P.); cauxudan@cau.edu.cn (D.X.); dwlai@cau.edu.cn (D.L.)

**Keywords:** histone acetylation modification, epigenetic regulation, histone acetytransferase, histone deacetylase, secondary metabolites, secondary metabolite biosynthesis, biosynthetic gene cluster, biological activities, regulation mechanisms

## Abstract

The histone acetylation modification is a conservative post-translational epigenetic regulation in fungi. It includes acetylation and deacetylation at the lysine residues of histone, which are catalyzed by histone acetyltransferase (HAT) and deacetylase (HDAC), respectively. The histone acetylation modification plays crucial roles in fungal growth and development, environmental stress response, secondary metabolite (SM) biosynthesis, and pathogenicity. One of the most important roles is to regulate the gene expression that is responsible for SM biosynthesis in fungi. This mini-review summarized the regulation of histone acetylation modification by HATs and HDACs on the biosynthesis of SMs in fungi. In most cases, histone acetylation by HATs positively regulated the biosynthesis of fungal SMs, while HDACs had their negative regulations. Some HATs and HDACs were revealed to regulate fungal SM biosynthesis. Hda1 was found to be the most efficient regulator to affect the biosynthesis of SMs in fungi. The regulated fungal species were mainly from the genera of *Aspergillus*, *Calcarisporium*, *Cladosporium*, *Fusarium*, *Monascus*, *Penicillium*, and *Pestalotiopsis*. With the strategy of histone acetylation modification, the biosynthesis of some harmful SMs will be inhibited, while the production of useful bioactive SMs will be promoted in fungi. The subsequent research should focus on the study of regulatory mechanisms.

## 1. Introduction

**Figure 1:**
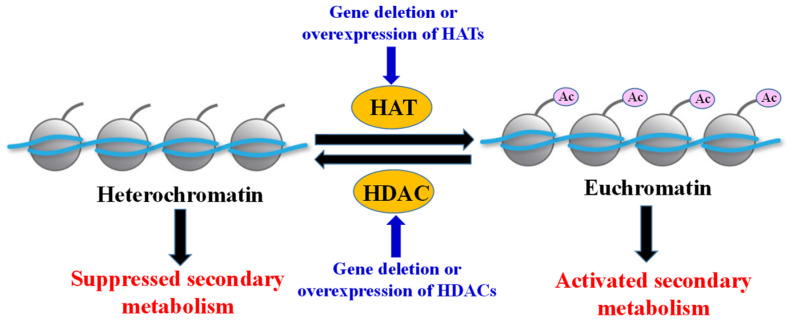
Histone acetylation modification regulated by histone acetyltransferases (HATs) and histone deacetylases (HDACs) to affect biosynthesis of SMs in fungi [11,12].

The histone acetylation modification is also called histone posttranslational acetylation modification, which includes acetylation and deacetylation at the lysine residues of histone. Multiple histone acetyltransferases (HATs) and histone deacetylases (HDACs) maintain a dynamic equilibrium of histone acetylation. HATs catalyze the transfer of acetyl groups from acetylcoenzyme A onto the ε-amino group on the side chain of lysine residues of core histones and commonly form a part of complexes. On the contrary, HDACs remove acetyl moieties from lysine residues at histone tails and nuclear regulatory proteins and thus significantly impact chromatin remodeling and transcriptional regulation in eukaryotes (Figure 1). So, HATs and HDACs are also called lysine acetyltranferases (KATs) and lysine deacetylases (KADCs), respectively [1,2,3].

Yeasts are important model organisms for studying histone acetylation. The functions and mechanisms of the most HATs and HDACs in fungi were originally discovered in yeasts. The studies on the model yeasts *Saccharomyces cerevisiae*, *Schizosaccharomyces pombe*, and *Candida albicans* contributed enormously to elucidate the landscape of eukaryote histone acetylation modification, which offered us an excellent entry point for gaining insights into these two types of enzymes [4,5,6].

DNA in chromatin is organized in arrays of nucleosomes. Two copies of each histone protein subunit, including H2A, H2B, H3, and H4, are assembled into an octamer that has 145–147 base pairs of DNA wrapped around it to form a nucleosome core [7]. There are about 30 acetylation sites, including H2AK5, H2AK9, H2AK13, H2AK15, and H2AK36 for histone H2A; H2BK5, H2BK11, H2BK12, H2BK15, H2BK16, H2BK20, and H2BK116 for histone H2B; H3K4, H3K9, H3K14, H3K18, H3K23, H3K27, H3K36, H3K37, H3K56, and H3K79 for histone H3; and H4K5, H4K8, H4K12, H4K16, H4K20, H4K31, H4K79, and H4K91 for histone H4 [8,9].

The histone acetylation level is maintained by HATs and HDACs. The histone acetylation modification is able to determine whether the chromatin is tightly packed together or becomes loosely packed, thereby controlling the regulation of transcriptional activation of DNA-binding proteins. It also occurs that this may bring genes closer to or away from transcription factors that continue to affect gene expression. The transcriptional regulation of gene expression is dependent on the state of chromatin, and chromatin competence itself is dependent on the epigenetic regulation of histone acetylation modification [6]. Both HATs and HDACs govern diverse biological processes, such as transcription activation, gene silencing, DNA replication, vegetative growth, asexual sporulation, sexual reproduction, stress response, cell cycle regulation, secondary metabolite (SM) biosynthesis, virulence, and pathogenicity of fungi [10].

In the past 20 years, many advances have been achieved in the regulation of histone acetylation modification on fungal secondary metabolism for either unlocking SMs or inhibiting SM production through fungal genomes [11,12,13]. However, specific reviews about the regulations of histone acetylation modification on SM biosynthesis in fungi have not been reported. In this mini-review, we focused on the regulation of HATs and HDACs on the biosynthesis of SMs in fungi in order to either inhibit the production of harmful metabolites or promote the production of useful bioactive metabolites. On the other hand, this mini-review attempts to elucidate their regulation mechanisms and accelerate their applications. The histone acetylation modification regulated by HATs and HDACs to affect the biosynthesis of SMs in fungi is shown in Figure 1.

## 2. Regulation of HATs in Biosynthesis of SMs in Fungi

Histone acetyltransferases (HATs) catalyze the acetylation of lysine residues within the *N*-terminal tails and globular domains of histones by transferring an acetyl group and using acetyl-CoA as a substrate. By this modification, the positive charges of basic lysine residues were neutralized, thereby reducing the affinity between histones and negatively charged DNA strands. The heterochromatin was then transformed into the transcriptionally active euchromatin, enabling gene expression (Figure 1). Therefore, histone acetylation by HATs usually positively regulates the biosynthesis of fungal SMs [14,15].

The HATs are classified into two major types (i.e., types A and B) based on their cellular locations and functions. Type A HATs are mainly located in both the nucleus and acetylated nucleosomal histones, whereas type B HATs are placed in both cytoplasm and acetylated newly synthesized histones. Moreover, based on the conserved structural motifs, type A HATs can be further divided into five categories, which are the GNAT (Gcn5-related *N*-acetyltransferases) family (i.e., Elp3, Gcn5, hat1, and Hpa2), MYST family (i.e., EsaA, Mst2, MystA, SAS3, and SAS2), p300/CBP (CREB-binding protein) family (i.e., Rtt109), basal transcription factors, and nuclear receptor co-activators. Compared to the GNAT family, the roles of the MYST family remain to be largely understood. In the p300/CBP family, only Rtt109 has been found in fungi [16,17,18,19]. The examples of HATs regulating SM production in fungi are shown in Table 1.

**Table 1:** The examples of HATs regulating SM production in fungi.

HAT	Fungus	Overexpression /Deletion	Positive/Negative Regulation	Production of SMs	Ref.
GNAT family					
AdaB					
	*Aspergillus* *nidulans*	Deletion	Positive	Reduced production of orsellinic acid (**1**) and lecanoric acid (**2**)	[20]
	*A. nidulans*	Overexpression	Positive	Induced production of orsellinic acid (**1**), lecanoric acid (**2**), F-9775A (**3**), and F-9775B (**4**).	[20]
Ada3					
	*Monascus* *ruber*	Deletion/Overexpression	Positive	Reduced pigment production in deletion mutant and increased pigment production in overexpressed mutant	[21]
	*M. ruber*	Deletion/Overexpression	Negative	Increased citrinin (**5**) production in deletion mutant and decreased citrinin (**5**) production in overexpressed mutant.	[21]
Elp3					
	*A. fumigatus*	Deletion	Positive	Decreased production of gliotoxin (**6**)	[19]
	*Fusarium* *graminearum*	Deletion	Negative	Decreased production of trichothecenes, including deoxynivalenol (**7**) and 15-acetyldeoxynivalenol (**8**)	[22]
Gcn5					
	*A. fumigatus*	Deletion	Positive	Inhibited biosynthesis of glutamine (**9**)	[23]
	*A. fumigatus*	Deletion	Negative	Increased production of several metabolites, including gliotoxin (**6**)	[19]
	*A. niger*	Deletion	Negative	Induced production of one novel compound nigerpyrone (**10**) and five known compounds carbonarone A (**11**), pestalamide A (**12**), funalenone (**13**), aurasperone A (**14**), and aurasperone E (**15**)	[24]
	*F. graminearum*	Deletion	Positive	Decreased production of DON (**7**)	[25,26,27]
	*M. ruber*	Deletion	Positive	Reduced production of citrinin (**5**)	[28]
Hat1					
	*Chaetomium* *globosum*	Overexpression	Positive	Induced production of azaphilones (**16**–**23**)	[29]
	*F. fujikuroi*	Deletion/Overexpression	Positive	Decreased production of GAs in deletion mutant and increased production of GAs in overexpressed mutant.	[30]
	*Metarhizium* *robertsii*	Deletion	Negative	Induced production of eight isocoumarin derivatives meromusides A–H (**24**–**31**), one isobenzofuranone derivative named meromuside I (**32**), and two nonribosomal peptides meromutides A (**33**) and B (**34**)	[31]
	*Monosporascus* *eutypoides*	Overexpression	Positive	Induced production of two new compounds, monosporasols A (**35**) and B (**36**), and two known compounds, pestaloficin C (**37**) and arthrinone (**38**)	[32]
	*Pestalotiopsis* *microspora*	Deletion	Positive	Reduced production of pestalotiollide B (**39**) and other SMs	[33]
MystB					
	*A. flavus*	Deletion	Positive	Decreased production of aflatoxin B1 (**40**)	[34]
NnaB					
	*A. nidulans*	Deletion	Positive	Induced production of known orsellinic acid derivatives, including orsellinic acid (**1**), lecanoric acid (**2**), diorcinol (**41**), cordyol C (**42**), violaceol I (**43**), and violaceol II (**44**), as well as the newly heterocyclic products pheofungins A–D (**45**–**48**)	[35]
SptJ					
	*Chaetomium* *globusum*	Deletion	Negative	Induced production of mollipilins A (**49**) and B (**50**), coarctatin (**51**), and dihydrocoarctatin (**52**)	[36]
**MYST family**					
EsaA					
	*A. fumigatus*	Overexpression	Positive	Increased production of orsellinic acid (**1**), sterigmatocystin (**53**), penicillin G (**54**), and terrequinone A (**55**)	[37]
	*M. ruber*	Overexpression	Positive	Increased production of polyketides including azaphilone pigments and citrinin (**5**)	[38]
Mst2					
	*P. microspora*	Deletion	Positive	Decreased production of pestalotiollide B (**39**)	[39]
	*P. microspora*	Overexpression	Negative	Decreased production of pestalotiollide B (**39**)	[39]
MystA					
	*A. flavus*	Deletion	Negative	Increased production of aflatoxin B1 (**40**)	[34]
Sas3					
	*F. graminearum*	Deletion	Positive	Decreased production of DON (**7**)	[26]
**p300/CBP family**					
Rtt109					
	*A. flavus*	Deletion	Positive	Decreased production of aflatoxin B1 (**40**)	[40]
	*M. purpureus*	Deletion	Negative	Increased production of citrinin (**5**) and pigments	[41]

### 2.1. Regulation of GNAT Family HATs on Biosynthesis of SMs in Fungi

GNAT (Gcn5-related *N*-acetyltransferases) family HATs have high similarity with yeast Gcn5, which is functioned in the general regulation of amino acid synthesis signaling pathway and exhibits transcriptional-associated HAT. Its biological activity seems to be dependent on the association in different multi-subunit complexes, such as the SAGA complex (Spt-Ada-Gcn5-Acetyltransferase) and the ADA complex (Ada2-Gcn5-Ada3). Some HATs in the GNAT family, including AdaB, Ada3, Elp3, Gcn5, hat1, MystB, NnaB, and SptJ, have been revealed to regulate the biosynthesis of SMs in fungi (Table 1).

#### 2.1.1. Regulation of AdaB

The deletion of *adaB*, the gene encoding histone acetyltransferase in *Aspergillus nidulans* during co-cultivation with *Streptomyces rapamycinicus*, led to reduced production of orsellinic acid (**1**) and lecanoric acid (**2**). Further investigation showed that Saga/Ada complex-mediated histone acetylation triggered the expression of the *ors* genes and the formation of orsellinic acid (**1**), lecanoric acid (**2**), F-9775A (**3**), and F-9775B (**4**) with their structures, as shown in Figure S1 [20].

#### 2.1.2. Regulation of Ada3

The *Mrada3* gene played a vital role in the sporulation development and secondary metabolism of *M. ruber*. The deletion of *Mrada3* in *M. ruber* resulted in slower growth, decreased pigment production, and increased citrinin (**5**) (Figure S1) production during the late period of fermentation. However, for the *Mrada3*-overexpressed strain, the number of ascospores and the pigment content were significantly higher than those of the wild-type (WT) strain, but citrinin (**5**) yield was slightly lower than that of the WT strain. It indicated that Ada3 positively regulated pigment production and negatively regulated citrinin (**5**) production in *M. ruber* [21].

#### 2.1.3. Regulation of Elp3

Elp3 is an abbreviation of elongator complex protein 3. In *Aspergillus fumigatus*, Elp3 played the opposite role to GcnE/Gcn5 in the production of SMs. The Δ*Elp3* mutant exhibited decreased gliotoxin (GT, **6**) compared to the WT strain. The production of numerous unidentified metabolites was also decreased in the strain without *Elp3* compared to the WT strain [19].

In the *Elp3* deletion mutant of *F. graminearum*, the number of perithecia was reduced and the maturation of perithecia was delayed. The main trichothecenes, such as deoxynivalenol (DON, **7**) and 15-acetyldeoxynivalenol (**8**), were not detected in the *Elp3* deletion mutant, whereas both compounds (**7** and **8**) could be produced at detectable levels in the wild-type strain. The RT-qPCR analysis showed that the transcription of the trichothecene biosynthesis genes *tri5* and *tri6* was significantly reduced in the *Elp3* deletion mutant compared with the WT strain. In a virulence test, 21 days after wheat head inoculation, the *Elp3* deletion mutant caused significantly reduced disease symptoms, whereas the WT and complementary strains caused typical head blight symptoms, which indicated that *Elp3* was involved in diverse biological processes, including sexual and asexual reproduction, SM production, and the virulence of *F. graminearum* [22]. The structures of the compounds produced by the fungi regulated by Elp3 are shown in Figure S1.

#### 2.1.4. Regulation of Gcn5

Gcn5 or GcnE (general control non-depressible 5) is a HAT in the SAGA/ADA complex. GcnE is involved in elevated levels of histone H3K9 acetylation. The target genes of GcnE include those involved in fungal secondary metabolism and development. It plays a critical role in the epigenetic landscape and chromatin modification for regulating a wide variety of biological events, including fungal SM biosynthesis and development [25]. The structures of the compounds produced by the fungi regulated by GcnE are shown in Figure S1.

The Δ*AflgcnE* mutant did not produce aflatoxins, which was consistent with a significant downregulation of aflatoxin gene expression levels in *A. flavus* [42].

The dual functions of GcnE in *A. fumigatus* were revealed for controlling the biosynthesis of glutamine (**9**) [23]. The Δ*GcnE* mutant of *A. fumigatus* showed an increased production of several metabolites, including gliotoxin (GT, **6**) [19].

The deletion of the *GcnE* gene resulted in the activation of the production of polyketides, including one novel compound nigerpyrone (**10**) and five known compounds carbonarone A (**11**), pestalamide A (**12**), funalenone (**13**), aurasperone A (**14**), and aurasperone E (**15**) in *A. niger* FGSC A1279. The results indicated that histone acetylation modification was an important strategy in activating the biosynthesis of SMs in *A. niger* [24].

Deoxynivalenol (DON, **7**) is a mycotoxin produced by *Fusarium* species. This mycotoxin is a virulence factor that helps fungi colonize and spread within spikes. DON (**7**) was originally called vomitoxin to have a critical emesis effect in some animals, such as humans, pigs, dogs, and minks [43]. The deletion of *GcnE* in *F. graminearum* led to the decreased production of DON (**7**) [25,26,27].

The contamination of citrinin (**5**) in Hongqu (*M. ruber*) has caused controversy about its safety. So, the control of citrinin (**5**) has always been an important issue of Hongqu products. The citrinin (**5**) content in the MrGcn5 null strain was dramatically reduced in the late growth period. Even the citrinin (**5**) content was as low as 21% in the wild-type strain, and RT-qPCR analysis showed that the expression level of genes involved in citrinin synthesis was significantly attenuated in the MrGcn5 null strain. So, MrGcn5 was very suitable for targeted improvement to reduce citrinin production in the fermentation process of Hongqu [28].

#### 2.1.5. Regulation of Hat1

The overexpression of histone acetyltransferase gene *hat1* in *Chaetomium globosum* induced production of two new dimeric bis-spiro-azaphilones named cochliodones A (**16**) and B (**17**), along with six known azaphilones chaetomugilin A (**18**), chaetomugilin D (**19**), 11-*epi*-chaetomugilin A (**20**), chaetoviridin A (**21**), chaetoviridin F (**22**), and chaetoviridin H (**23**). The RT-qPCR analysis also revealed that one biosynthetic gene cluster (BGC) was activated to produce a series of azaphilone analogs [29].

The deletion of *hat1* in *Fusarium fujikuroi* resulted in the downregulation of gibberellin (GA) biosynthesis gene expression and decreased GA production. The overexpression of *hat1* resulted in the upregulation of GA gene expression and increased GA production [30].

The deletion of *hat1* in *Metarhizium robertsii* led to the characterization of 11 new SMs, including eight isocoumarin derivatives meromusides A (**24**), B (**25**), C (**26**), D, (**27**), E (**28**), F (**29**), G (**30**), and H (**31**); one isobenzofuranone derivative with a substituted benzene ring attached at the C-3 position of the furanone ring named meromuside I (**32**); and two nonribosomal peptides named meromutides A (**33**) and B (**34**). Among 12 polyketide synthase (PKS) genes, the relative expressions of six PKS genes were significantly up-regulated by RT-qPCR analysis. Therefore, this confirmed that hat1 played a negative regulation role in the gene expression as a rare case. The disruption of HAT represented a new approach to mine chemical diversity in fungi [31].

The overexpression of the *Mehat* gene in *Monosporascus eutypoides* induced the production of new metabolites, monosporasols A (**35**) and B (**36**), and two known SMs, pestaloficin C (**37**) and arthrinone (**38**). Moreover, the ∆*hat1* mutant delayed the production of conidia with a significantly decreased number of conidia. The integrity of the cell wall was also impaired in the ∆*hat1* mutant [32].

The deletion of *hat1* in the taxol-producing fungus *Pestalotiopsis microspora* NK17 reduced the production of pestalotiollide B (**39**) and other SMs [33]. The structures of the compounds produced by the fungi regulated by hat1 are shown in Figure S1.

#### 2.1.6. Regulation of MystB

MystB acetylated H3K14, H3K18, and H3K23 into H3K14ac, H3K18ac, and H3K23ac, respectively, in the nucleus. The deletion of *mystB* in *A. flavus* led to significant defects in conidiation, sclerotia formation, and aflatoxin B1 (**40**) (Figure S1) production [34].

#### 2.1.7. Regulation of NnaB

The deletion of *nnaB*, a GNAT-type histone acetyltransferase-encoding gene, induced the production of six known orsellinic acid derivatives, orsellinic acid (**1**), lecanoric acid (**2**), diorcinol (**41**), cordyol C (**42**), violaceol I (**43**), and violaceol II (**44**), as well as four newly heterocyclic metabolites, pheofungins A (**45**), B (**46**), C (**47**), and D (**48**), in *A nidulans*. The structures of compounds **41**–**48** are shown in Figure S1. Among these metabolites, pheofungin C (**47**) effectively inhibited the proliferation of HUVEC and K-562 human leukemia cell lines. Furthermore, pheofungin C (**47**) showed cytotoxic effects against HeLa cells with an IC_50_ value of 10 μM. The detailed regulation mechanism of NnaB indicated that the histone repressor function required acetylation by NnaB. The loss of NnaB activity led to a global stress reaction as the *N*-terminal acetylation of histones was essential for the functions of cell processes. The production of orsellinic acid derivatives and phenofungins was the fungal response to global stress caused by impaired posttranslational modification [35].

#### 2.1.8. Regulation of SptJ

CgSptJ in *Chaetomium globusum* was a HAT homolog of *S. cerevisiae* Spt10 that could install a chromatin mark associated with gene silencing and heterochromatin formation. *CgSptJ* deletion induced the production of mollipilins A (**49**) and B (**50**), coarctatin (**51**), and dihydrocoarctatin (**52**) in *C. globusum* [36]. The structures of compounds **49**–**52** are shown in Figure S1.

### 2.2. Regulation of MYST Family HATs on Biosynthesis of SMs in Fungi

MYST family HATs are conserved from yeast to humans and are involved in diverse biological functions, including gene regulation, DNA repair, and cell-cycle regulation and development. Some HATs in the MYST family, including EsaA, Mst2, MystA, and Sas3, have been revealed to regulate the biosynthesis of SMs in fungi (Table 1).

#### 2.2.1. Regulation of EsaA

The overexpression of histone 4 lysine 12 (H4K12) acetyltransferase EsaA increased the production of SMs, including orsellinic acid (**1**), sterigmatocystin (**53**), penicillin G (**54**), and terrequinone A (**55**) (Figure S1) in *A. nidulans* [37].

The azaphilone pigments (MonAzPs) and citrinin (**5**) production of the *Mresa1*-overexpressed strain of *M. ruber* were 1.7 and 2.4 times more than those of the wild-type (WT) strain, respectively. The overexpression of *Mresa1* accelerated growth and increased ascospores yield and polyketide production in *M. ruber* [38].

#### 2.2.2. Regulation of Mst2

Pestalotiollide B (PB, **39**) production was diminished 7-fold in the Δ*mst2* mutant of *Pestalotipsis microspora* NK17. However, the overexpression of *mst2* also resulted in an overall reduction of PB (**39**) production in the *mst2* overexpression mutant [39].

#### 2.2.3. Regulation of MystA

MystA was located in the nuclei and cytoplasm, and MystA could acetylate H4K16ac. The deletion of *MystA* in *A. flavus* resulted in decreased conidiation and increased sclerotia formation and aflatoxin B1 (**40**) production, which meant that MystA negatively regulated aflatoxin B1 (**40**) biosynthesis [34].

#### 2.2.4. Regulation of Sas3

Sas3 (or SasC) governs diverse biological processes in fungi. It has been considered a potential target for controlling pathogenic fungi. The deletion of *sasC* in the opportunistic human pathogenic fungus *A. fumigatus* resulted in drastically reduced colony growth, asexual development, spore germination, response to stresses, and fungal virulence. Western blot analysis showed that SasC likely catalyzed the acetylation of H3K9, K3K14, and H3K29 in *A. fumigatus* [44].

The deletion of *FgSas3* decreased the production of deoxynivalenol (DON, **7**) in *F. graminearum*. Additionally, ∆*FgSas3* showed reduced sporulation and perithecium formation [26].

### 2.3. Regulation of p300/CBP Family HATs on Biosynthesis of SMs in Fungi

The p300/CBP (CREB-binding protein) family belongs to type A HATs. Rtt109 (regulator of Ty1 transposition gene product 109), also known as KAT11, is a lysine acetyltransferase that usually acetylates histone H3 at lysine 56 (H3K56) in the p300/CBP family [45]. So far, only Rtt109 was found in fungi (Table 1). This acetylation event is important for proper DNA replication and repair to occur.

The amount of aflatoxin B1 (**40**) synthesized in the Δ*Rtt109* mutant of *A. flavus* was significantly decreased in the PDB liquid medium. It was found that H3K9 was the acetylated target of Rtt109 in *A. flavus*. Through the maize seed infection experiment, the growth of the Δ*Rtt109* mutant on the surface of affected corn was largely reduced, and the amount of aerial mycelium decreased significantly, which was consistent with the results of the artificial medium. This indicated that Rtt109 participated in aflatoxin synthesis and the regulation of infection of *A. flavus* [40].

The deletion of *Rtt109* in *Monascus purpureus* significantly reduced conidia formation and colony growth, whereas it increased the yield of citrinin (CTN, **5**) and pigments. RT-qPCR analysis indicated that Rtt109 remarkably affected the transcriptional expression of key genes related to the development, morphogenesis, and secondary metabolism of *M. purpureus* [41].

## 3. Regulation of HDACs on Biosynthesis of SMs in Fungi

Histone deacetylases (HDACs) catalyze the removal of the acetyl moiety from acetyl-lysine residues within the *N*-terminal tails and globular domains of histones to promote gene repression and silencing (Figure 1). Therefore, histone deacetylation by HDACs usually negatively regulates the biosynthesis of fungal SMs [11,12].

According to the classification rule of HDACs in yeast, fungal HDACs are mainly categorized into three classes (i.e., classes I, II, and III). Both class I and class II are Zn^2+^-dependent HDACs, and class III is a NAD^+^-dependent HDAC. Class I is also called the Rpd3 family, which includes Hos2 (HDA one similar 2) and Rpd3 (reduced potassium dependency 3). Class II is also called the Hda1 family, which contains Hda1 (histone deacetylase 1) and Hos3. Class III (also called sirtuin family) includes Sir2 (silent information regulator 2) and Hst1–4 (homologs of Sir2) [5,46]. The examples of HDACs regulating SM production in fungi are shown in Table 2.

**Table 2:** The examples of HDACs regulating SM production in fungi.

HDAC	Fungus	Overexpression /Deletion	Positive/Negative Regulation	Production of SMs	Ref.
Class I HDAC					
Hda2					
	*F. fujikuroi*	Deletion	Positive	Decreased production of GA3 (**56**), GA4 (**57**), GA7 (**58**), bikaverin (**59**), fusarubin (**60**), and fusaric acid (**61**)	[47]
HosA					
	*A. flavus*	Deletion	Positive	Decreased production of AFB1 (**40**)	[48]
	*A. nidulans*	Deletion	Positive	Decreased production of penicillin G (**54**)	[49]
	*A. nidulans*	Deletion	Negative	Increased production of orsellinic acid (**1**), aspercryptin (**62**), and cichorine (**63**)	[49]
	*A. niger*	Deletion	Positive	Decreased production of fumonisin B1 (**64**) and slightly increased production of kojic acid (**65**)	[50]
Hos2					
	*Chaetomium* *olivaceum*	Deletion	Negative	Increased production of orsellinic acid (**1**), globosumone C (**66**), orsellide A (**67**), and cochliodinol (**68**)	[51]
	*A. nidulans*	Deletion	Negative	Increased production of sterigmatocystin (**53**) and penicillin G (**54**)	[52]
	*F. verticillioides*	Deletion	Positive	Decreased fumonisin B1 (**64**) production	[53]
	*M. ruber*	Deletion/Overexpression	Positive	Dramatically decreased production of pigments in ∆*Mrhos2* mutant, while overexpression of *Mrhos2* significantly increased production of pigments	[54]
	*Ustilaginoidea* *virens*	Deletion	Positive	Reduced production of ustilaginoidins and sorbicillinoids	[55]
RpdA					
	*A. flavus*	Deletion	Positive	Lost production of aflatoxin B1 (**40**)	[56,57]
	*A. nidulans*	Deletion	Negative	Increased production of alternariol (**69**), F 9775A (**70**) F 9775B (**71**), and three fellutamides, fellutamide B (**72**), antibiotic 1656G (**73**), and antibiotic 3127 (**74**)	[58]
	*A. nidulans*	Deletion	Negative	Increased production of two lipopeptides aspercryptins A1 (**75**) and A2 (**76**)	[59]
Rpd3					
	*F. verticillioides*	Overexpression	Positive	Increased production of fumonisin B1 (**64**)	[53]
	*M. ruber*	Overexpression	Positive	Increased production of citrinin (**5**)	[60]
Rxt3					
	*A. oryzae*	Deletion	Negative	Increased production of kojic acid (**65**)	[61]
**Class II HDAC**					
Clr3					
	*P. brasilianum*	Deletion	Positive	Reduced production of 14 SMs, austin-related meroterpenoids (**77**–**80**), brasiliamides (**81**–**85**), verruculogen (**86**), verruculogen TR2 (**87**), penicillic acid (**88**), and cyclodepsipeptides JBIR 114 (**89**) and JBIR 115 (**90**)	[62]
HdaA					
	*A. flavus*	Deletion	Positive	Decreased production of AFB1 (**40**)	[48]
	*A. fumigatus*	Deletion	Positive	Decreased production of gliotoxin (**6**)	[63]
	*A. nidulans*	Deletion	Negative	Increased production of sterigmatocystin (**53**) and penicillin G (**54**)	[52]
	*A. niger*	Deletion	Positive	Decreased production of fumonisin B1 (**64**) and kojic acid (**65**)	[50]
	*A. terreus*	Deletion	Negative	Increased production of butyrolactones I (**91**) and II (**92**), and induced production of two new azaphilones asperterilones A (**93**) and B (**94**)	[64]
	*Calcarisporium* *arbuscula*	Deletion	Negative	Overproduced ten compounds including three cyclopeptides, namely arbumycin (**95**), arbumelin (**96**), and verlamelin A (**97**); three polyketides named sterigmatocystin (**53**) and paeciloquinones A (**98**) and B (**99**); three diterpenoids named zythiostromic acids A (**100**) and B (**101**) and arbusculic acid A (**102**); and one meroterpenoid arbuscullic acid B (**103**)	[65]
	*C. arbuscula*	Deletion	Negative	Induced production of fourteen diterpenoids including three cassanes named calcarisporic acids A–C (**104**−**106**), one cleistanthane named calcarisporic acid D (**107**), six pimaranes named calcarisporic acids E−J (**108**−**113**), two isopimaranes named calcarisporic acids K (**114**) and L (**115**), and two cleistanthanes named hawiinolide G (**116**) and 14-*epi*-zythiostromic acid B (**117**)	[66]
	*C. fulvum*	Overexpression/Deletion	Positive	Induced production of cladofulvin (**118**) in overexpression mutant and abolished production of cladofulvin (**118**) in deletion mutant	[67,68]
	*F. fujikuroi*	Deletion	Negative	Increased production of beauvericin (**119**)	[69]
	*F. fujikuroi*	Deletion	Positive	Decreased production of GA3 (**56**), GA4 (**57**), GA7 (**58**), bikaverin (**59**), fusarubin (**60**), and fusaric acid (**61**)	[47]
	*F. fujikuroi*	Deletion	Negative	Increased production of fusarin A (**120**)	[47]
	*F. verticillioides*	Deletion	Negative	Increased production of fumonisin B1 (**64**)	[53]
	*Magnaporthe* *oryzae*	Deletion	Negative	Increased production of ergosterol (**121**) and 1,8-dihydroxynaphthalene derivatives including 3,4,8-trihydroxytetralone (**122**), 4,6,8-trihydroxytetralone (**123**) and 4,8-dihydroxytetralone (**124**)	[70]
	*P. chrysogenum*	Deletion	Negative	Increased production of sorbicillinoids including sorbicillin (**125**), 2′,3-dihydrosorbicillin (**126**), sorbicillinol (**127**), 2′,3′-dihydrosorbicillinol (**128**), oxosorbicillinol (**129**), bisorbicillinol (**130**), bisvertinolone (**131**), dihydrobisvertinolone (**132**), and tetrahydrobisvertinolone (**133**), as well as nitrogen-containing compounds chrysogine (**134**), *N*-acetylalanylanthranilamide (**135**), and *N*-pyrovoylanthranilamid (**136**)	[71]
	*P. chrysogenum*	Deletion	Negative	Increased production of meleagrin (**137**), roquefortine F (**138**), and roquefortine C (**139**)	[72]
	*P. chrysogenum*	Deletion	Positive	Decreased production of chrysogine (**134**)	[72]
	*Pestalotiopsis* *fici*	Deletion	Negative	Induced production of 12 new polyketides including macrodiolide ficiolides A–K (**140**–**150**), pestaloficiol W (**151**), and known polyketide asperpentyn (**152**)	[73]
	*P. fici*	Deletion	Negative	Induced production of one novel compound, namely pestaloficiol X (**153**), and seven known compounds, including pestaloficiol M (**154**), pestaloficin D (**155**), isosulochrin (**156**), chloropupukeananin (**157**), pestaloficiol J (**158**), hydroxyisoseiridin (**159**), and pestheic acid (**160**)	[74]
Hdf1					
	*F. graminearum*	Deletion	Positive	Decreased production of DON (**7**)	[75]
Hdf2					
	*F. asiaticum*	Deletion	Negative	Increased production of 4-ANIV (**161**) and 4,15-diANIV (**162**)	[70]
Hid1					
	*P. microspora*	Deletion	Negative	Increased production of pestalotiollide B (**39**)	[76]
Hos3					
	*M. ruber*	Deletion	Negative	Increased production of citrinin (**5**) and pigments including ankaflavin (**163**), monascin (**164**), monasfluore A (**165**), monasfluore B (**166**), monascorubrin (**167**), and rubropunctatin (**168**)	[77]
**Class III HDAC**					
Hst2					
	*F. verticillioides*	Deletion	Negative	Increased production of fumonisin B1 (**64**)	[53]
Hst4					
	*A.* *oryzae*	Deletion	Negative	Increased production of kojic acid (**65**)	[78]
	*A. terreus*	Deletion	Positive	Decreased production of butyrolactones I (**91**) and II (**92**) and lovastatin (**169**)	[79]
	*A. terreus*	Deletion	Negative	Increased production of terrein (**170**)	[79]
	*M. ruber*	Deletion	Negative	Increased production of citrinin (**5**) and azaphilone, including ankaflavin (**163**), monascin (**164**), monasflore A (**165**), monasflore B (**166**), monascuburin (**167**), and rubropunctatin (**168**)	[80]
SirA					
	*A.* *nidulans*	Deletion	Negative	Increased production of sterigmatocystin (**53**) and penicillin G (**54**). Dihydrocoumarin was used to treat *M*. *ruber* to increase production of monasfluol B (**171**), acetyl monasfluol B (**172**), and monascusazaphilone C (**173**), while inhibiting production of citrinin (**5**)	[81,82]
	*A.* *nidulans*	Deletion	Negative	Increased production of sterigmatocystin (**53**) and austinol (**79**)	[83]
Sir2					
	*F. verticillioides*	Deletion	Negative	Increased production of fumonisin B1 (**64**)	[53]
	*Metarhizium* *robertsii*	Deletion	Positive	Reduced content of ergosterol (**121**)	[84]
SirD					
	*A.* *kawachii*	Deletion	Positive	Decreased production of citric acid (**174**)	[85]
	*F. verticillioides*	Overexpression	Negative	Decreased production of fumonisin B1 (**64**)	[53]
SirE					
	*A.* *nidulans*	Deletion	Positive	Decreased production of sterimatocystin (**53**)	[86]
	*A.* *flavus*	Deletion	Negative	Increased production of aflatoxin B1 (**40**)	[87]
	*A. fumigatus*	Deletion	Negative	Increased production of pseurotin A (**175**), brevinamide F (**176**), pyripyroropene A (**177**), deacetyl pyriopyropene (**178**), and bis(methylthio)gliotoxin (**179**)	[88]

### 3.1. Regulation of Class I HDACs on Biosynthesis of SMs in Fungi

Class I HDACs that regulate fungal secondary metabolism include Hda2, HosA, Hos2, RpdA, Rpd3, and Rxt3 (Table 2).

#### 3.1.1. Regulation of Hda2

The deletion of the gene *ffhda2* in *F. fujikuroi* reduced the production of gibberellins (GAs), including GA3 (**56**), GA4 (**57**), and GA7 (**58**); bikaverin (BIK, **59**); fusarubin (FSR, **60**); and fusaric acid (FU, **61**), with their structures shown in Figure S1. It was estimated that FfHda2 was required for the virulence of *F. fujikuroi* by participating in the regulation of SM biosynthesis in rice seedlings [47].

#### 3.1.2. Regulation of HosA

HosA was a major regulator of secondary metabolism in fungi. The deletion of the HosA-encoding gene led to decreased production of aflatoxin B1 (AFB1, **40**) in *A. flavus.* The chromatin immunoprecipitation experiments indicated that HosA was bound directly to AFB1 biosynthesis cluster genes to regulate their expression [48].

The deletion of *hosA* led to the decreased production of penicillin G (**54**) and increased production of orsellinic acid (**1**), aspercryptin (**62**), and cichorine (**63**) in *A. nidulans*. [49].

The deletion of *hosA* led to the decreased production of fumonisin B1 (**64**) and slightly increased production of kojic acid (**65**) in *A. niger* FGSC A1279 based on the metabolomics analysis [50]. By applying the epigenetic CRISPR/dCas9 system to regulate the expression of the secondary metabolic genes in *A. niger*, histone deacetylase HosA activated the expression of *fwnA* and accelerated the biosynthesis of melanin [89]. The structures of compounds produced by the fungi regulated by HosA are shown in Figure S1.

#### 3.1.3. Regulation of Hos2

The g7489 protein was homologous to Hos2 of the class I HDACs. The deletion of *g7489* in *Chaetomium olivaceum* SD-80A increased the expression of SM biosynthesis gene clusters (BGCs) and resulted in an increased production of four compounds, which were identified as orsellinic acid (OA, **1**), globosumone C (**66**), orsellide A (**67**), and cochliodinol (**68**), with their structures shown in Figure S1. Among these compounds, globosumone C (**66**) exhibited moderate acetylcholinesterase inhibitory activity with an IC_50_ value of 7.34 μΜ [51].

The deletion of *hos2* increased the production of sterigmatocystin (**53**) and penicillin G (**54**) in *A. nidulans* [52]. The deletion of *hos2* increased melanin production in *Colletotrichum gloeosporioides* [90].

Fumonisin B1 (**64**) production was decreased in the ∆*Fvhos2* strains of *F. verticillioides*, which meant that fumonisin B1 (**64**) biosynthesis was positively regulated by FvHos2. Accordingly, *FUM* genes, such as *FUM1*, *FUM8*, *FUM19*, and *FUM21*, displayed significantly reduced expressions in the ∆*Fvhos2* strains. However, an increased level of H4K16ac was observed in the deletion mutant of FvHos2 in *F. verticillioides* [53].

*Monascus azaphilone* pigments (MonAzPs) are beneficial SMs secreted by *Monascus* spp., which have great potential for use, mainly in the food industry. The inactivation of *Mrhos2* dramatically decreased pigment production in *M. ruber*, while overexpression of *Mrhos2* significantly increased pigment production [54].

The UvHos2-deletion mutants of *U. virens* exhibited retarded vegetative growth, reduced conidial production and germination, and attenuated virulence. UvHos2 positively regulated tolerance to various environmental stresses, including cell wall and cell membrane integrity and osmotic and oxidative stresses. UvHos2 reduced the acetylation levels of histone at multiple lysine sites, including H3K9, H3K14, H3K27, and H3K56. ChIP-PCR assays revealed that UvHos2-mediated H3K9 deacetylation regulated the expression of ustilaginoidin biosynthesis genes. Consistently, transcriptome analysis indicated that UvHos2 regulated the expression of the genes involved in secondary metabolism, mycelial growth, conidiogenesis, and pathogenicity, thereby controlling *U. virens* virulence and the biosynthesis of ustilaginoidins and sorbicillinoids [55].

#### 3.1.4. Regulation of RpdA

KdmB-EcoA-RpdA-SntB (KERS) is a nuclear chromatin-binding complex that contains the JARID1-type histone demethylase KdmB, a putative cohesion acetyltransferase EcoA, a class I histone deacetylase RpdA, and the PHD ring finger reader protein SntB in *A. nidulans*. KdmB and RpdA of the KERS complex were considered the key regulators for fungal development and secondary metabolism in *A. flavus*. Both KdmB and RpdA regulated H3K4me3 and H3K9me3 levels, while RpdA also acted on H3K14ac levels in nuclear extracts. Aflatoxin production was lost in the Δ*kdmB* and Δ*rpdA* mutants, which meant that both *kdmB* and *rpdA* were essential for aflatoxin B1 (**40**) production in *A. flavus* [56,57].

The deletion of *RpdA* led to the increased production of alternariol (**69**), F9775A (**70**), and F9775B (**71**), as well as the three fellutamides fellutamide B (**72**), antibiotic 1656G (**73**), and antibiotic 3127 (**74**) in *A. nidulans*. This was the first time fellutamides were isolated from the fungus *A. nidulans* with minimal bias at the metabolomic level [58].

The deletion of *RpdA* led to increased production of two lipopeptides aspercryptins A1 (**75**) and A2 (**76**) in *A. nidulans* [59]. The structures of compounds produced by the fungi regulated by RpdA are shown in Figure S1.

#### 3.1.5. Regulation of Rpd3

The overexpression of *FvRpd3* in *F. verticillioides* increased fumonisin B1 (**64**) production. Accordingly, *FUM* genes such as *FUM1*, *FUM8*, *FUM19*, and *FUM21* displayed significantly high expression in the *FvRpd3*-OE strains [53].

The *Mrrpd3*-overexpressing strain of *M. ruber* enhanced the citrinin (**5**) content by 61.9%, 56.5%, and 52.6% on the day 3, day 9, and day 11, respectively. The overexpression of *Mrrpd3* significantly increased the relative expression of citrinin biosynthetic pathway genes, including *pksCT*, *mrl1*, *mrl2*, *mrl4*, *mrl6*, and *mrl7*. However, the overexpression of *Mrrpd3* had no significant effect on pigment production [60].

#### 3.1.6. Regulation of Rxt3

Rxt3 was a subunit in the Rpd3L histone deacetylase complex that regulated development, stress tolerance, amylase production, and kojic acid (**65**) synthesis in *A. oryzae*. The deletion of *Rxt3* increased the production of kojic acid (**65**) and augmented tolerance to multiple stresses, including cell wall stress, cell membrane stress, endoplasmic reticulum stress, osmotic stress, and oxidative stress [61].

### 3.2. Regulation of Class II HDACs on Biosynthesis of SMs in Fungi

Class II HDACs that regulate fungal secondary metabolism include Clr3, HdaA, Hdf1, Hdf2, Hid1, and Hos3 (Table 2).

#### 3.2.1. Regulation of Clr3

The gene *clr3* encoded a class II histone deacetylase in *P. brasilianum* LaBioMMi 136. The deletion of *clr3* led to the reduced production of 14 SMs, including austin-related meroterpenoids, such as isoaustinone (**77**), acetoxydehydroaustin (**78**), austinol (**79**), and austinoneol A (**80**); brasiliamides A (**81**), B (**82**), C (**83**), D (**84**), and E (**85**); verruculogen (**86**) and verruculogen TR2 (**87**); penicillic acid (**88**); and cyclodepsipeptides JBIR 114 (**89**) and JBIR 115 (**90**), with their structures, as shown in Figure S1 [62].

#### 3.2.2. Regulation of HdaA

HdaA (Hda1) has been found to regulate the biosynthesis of SMs in many fungal species. The structures of compounds produced by the fungi regulated by HdaA are shown in Figure S1. The deletion of *hdaA* led to the decreased production of aflatoxin B1 (AFB1, **40**) in *A. flavus* [48].

The deletion of *hdaA* in *A. fumigatus* increased the production of several SMs but decreased production of gliotoxin (**6**), whereas the overexpression of *hdaA* increased the production of gliotoxin (**6**). Unfortunately, the related SMs have not been identified, except for gliotoxin (**6**) [63].

The deletion of *hdaA* in *A. nidulans* increased the production of sterigmatocystin (**53**) and penicillin G (**54**) [52].

The deletion of *hdaA* in *A. niger* FGSC A1279 led to an increase in pigment production in a liquid Czapek Dox (CD) medium. According to the metabolomic analysis, the production of fumonisin B1 (**64**) and kojic acid (**65**) was reduced in the ∆*hdaA* mutant [50].

The deletion of *hdaA* in the marine-derived fungus *A. terreus* RA2905 increased the production of butyrolactones I (**91**) and II (**92**) and induced two new azaphilones asperterilones A (**93**) and B (**94**). Both azaphilones (**93** and **94**) displayed moderate anti-*Candida* activities, with the MIC values ranging from 18.0 to 47.9 μM, and asperterilones A (**93**) exhibited significant cytotoxic activity against human breast cancer cell line MDA-MB-23. This study indicated that *hdaA* played essential and global roles in repressing SM gene expression in fungi, and *hdaA* deletion represented an efficient strategy to mine new compounds from *A. terreus* [64].

The deletion of *hdaA* in *Calcarisporium arbuscula,* a mushroom endophytic fungus, was found to activate pleiotropic and led to the overexpression of more than 75% of the biosynthetic gene clusters. The overproduced ten metabolites in the ∆*hdaA* mutant were identified as three cyclopeptides, namely arbumycin (**95**), arbumelin (**96**), and verlamelin A (**97**); three polyketides named sterigmatocystin (**53**) and paeciloquinones A (**98**) and B (**99**); three diterpenoids named zythiostromic acids A (**100**) and B (**101**) and arbusculic acid A (**102**); and one meroterpenoid arbuscullic acid B (**103**). Among them, arbumycin (**95**), arbumelin (**96**), and arbusculic acids A (**102**) and B (**103**) were new compounds [65]. Subsequently, fourteen diterpenoids, including three cassanes named calcarisporic acids A (**104**), B (**105**), and C (**106**); one cleistanthane named calcarisporic acid D (**107**); six pimaranes named calcarisporic acids E (**108**), F (**109**), G (**110**), H (**111**), I (**112**), and J (**113**); two isopimaranes named calcarisporic acids K (**114**) and L (**115**); and two cleistanthanes named hawiinolide G (**116**) and 14-*epi*-zythiostromic acid B (**117**), were identified in the *C. arbuscula* ∆*hdaA* mutant. Among them, compounds **104**–**115** were new diterpenoids. Calcarisporic acids B (**105**) and D (**107**) significantly inhibited the expression level of matrix metalloproteinases MMP1 and MMP2 to show their potential as an anti-metastatic agent for the treatment of human breast cancer [66].

The overexpression of *CfHdaA* induced the production of cladofulvin (**118**) in *Cladosporium fulvum* [67]. Conversely, the deletion of *CfHdaA* led to the abolished production of cladofulvin (**118**) [68]. It clearly showed that *CfHdaA* positively regulated cladofulvin (**118**) production.

Melanin is a pigmented polymer that protects fungal cells against oxidative stress, phagocytosis, and fungicides. It also modifies the host immune responses by reducing the susceptibility of melanized microbes to the host defense mechanisms [91]. It was found that the change in virulence of the ∆*Hda1* mutant of *Cryptococcus neoformans* might be due to its markedly reduced formation of capsule, melanin, and extracellular proteases, all of which are specifically required for the survival of microbes in the host [92].

The deletion of *Hda1* in *F. fujikuroi* led to enhanced production of beauvericin (**119**) with a 1000-fold increase [69].

Deletion of the *FfHda1* in*F. fujikuroi* gene inhibited the production of gibberellins (GAs) including plant hormones GA3 (**56**), GA4 (**57**) and GA7 (**58**); red polyketide pigments bikaverin (BIK, **59**), fusarubin (FSR, **60**) and fusaric acid (FU, **61**), but resulted in increased production of fusarin A (FUS, **120**). It was estimated that FfHda1 was required for virulence in the rice seedlings [47].

Deletion of *FvHda1* in *F. verticillioides* led to increased production of fumonisin B1 (**64**), which meant FvHda1 negatively regulated fumonisin biosynthesis. Additionally, the RT-qPCR revealed an increase in the expression of FUM1 in the Δ*FvHda1* mutants. [53].

Deletion of *MoHda1* in *M. oryzae* resulted in increased production of ergosterol (**121**) and 1,8-dihydroxynaphthalene derivatives including 3,4,8-trihydroxytetralone (3,4,8-THT, **122**), 4,6,8-trihydroxytetralone (4,6,8-THT, **123**) and 4,8-dihydroxytetralone (4,8-DHT, **124**). It was also found that deletion of *MoHda1* in *M. oryzae* increased the expression of melanin biosynthesis genes [70].

The deletion of *HdaA* in *P. chrysogenum* led to the increased production of sorbicillinoids, including sorbicillin (**125**), 2′,3-dihydrosorbicillin (**126**), sorbicillinol (**127**), 2′,3′-dihydrosorbicillinol (**128**), oxosorbicillinol (**129**), bisorbicillinol (**130**), bisvertinolone (**131**), dihydrobisvertinolone (**132**), and tetrahydrobisvertinolone (**133**), as well as three nitrogen-containing compounds chrysogine (**134**), *N*-acetylalanylanthranilamide (**135**), and *N*-pyrovoylanthranilamid (**136**) [71]. Many bioactive sorbicillinoids were found in fungi, including *P. chrysogenum* [93,94]. In order to effectively increase the production of sorbicillinoids, further detailed investigation is needed on their biosynthetic regulations.

The deletion of HdaA in *P. chrysogenum* Fes1701 led to the increased production of meleagrin/rogquefortine-related compounds, including meleagrin (**137**), roquefortine C (**138**), and roquefortine F (**139**), as well as the decreased production of chrysogine (**134**) [72].

*Pestalotiopsis fici* was an endophytic fungus isolated from the branches of *Camellia sinensis* (Theaceae). The deletion of *PfHdaA* in *P. fici* induced the production of 12 new polyketides, macrodiolide ficiolides A (**140**), B (**141**), C (**142**), D (**143**), E (**144**), F (**145**), G (**146**), H (**147**), I (**148**), J (**149**), and K (**150**) and pestaloficiol W (**151**), as well as the known polyketide asperpentyn (**152**) [73]. Subsequently, one novel compound pestaloficiol X (**153**), along with seven additional known compounds pestaloficiol M (**154**), pestaloficin D (**155**), isosulochrin (**156**), chloropupukeananin (**157**), pestaloficiol J (**158**), hydroxyisoseiridin (**159**), and pestheic acid (**160**), were isolated from the ∆*PfHdaA* mutant [74].

*Trichoderma atroviride* was applied in agriculture as a biostimulant and biological control agent against fungal pathogens that infested crop plants. The deletion of *Hda1* led to the enhanced production of SMs, including 2-methyl-butanol, 3-methyl-butanol, ethanol, and 3-octanone. Moreover, *Hda1* deletion affected the expression of several notable gene categories, such as polyketide synthases, transcription factors, and genes involved in the HOG MAPK pathway [95].

#### 3.2.3. Regulation of Hdf1

The deletion of *Hdf1* resulted in a significant reduction in virulence and deoxynivalenol (DON, **7**) production in *F. graminearum*. In addition, the ∆*Hdf1* mutant was more tolerant to H_2_O_2_ than the wild-type strain [75].

#### 3.2.4. Regulation of Hdf2

The production of trichothecenes 4-acetylnivalenol (4-ANIV, **161**) and 4,15-diacetylnivalenol (4,15-diANIV, **162**) (Figure S1) was increased in the ∆*Hdf2* mutant of *F. asiaticum* [70].

#### 3.2.5. Regulation of Hid1

In the Δ*Hid1* mutant of *P. mcirospora* NK17, the yield of pestalotiollide B (**39**) increased approximately 2-fold to 15.90 mg/L. Pestalotiollide B (**39**) has been considered to have the potential to develop as the inhibitor of cholesterol ester transfer protein (CETP) and acyl-CoA:cholesterol acyltransferase (ACAT). Moreover, the deletion of *Hid1* led to a dramatic decrease in conidia production in the fungus [76].

#### 3.2.6. Regulation of Hos3

*M. ruber* can produce a variety of beneficial metabolites used in food and pharmaceutical industries. However, this fungus can produce citrinin (**5**) with toxic effects on mammals [96]. The deletion of *Mrhos3* caused an enhancement of citrinin (**5**) content. Correspondingly, the relative expression of citrinin (**5**) biosynthetic pathway genes *pksCT*, *mrl1*, *mrl2*, *mrl4*, *mrl6*, and *mrl7* was increased. In addition, the deletion of *Mrhos3* led to increased production of edible pigments, including yellow pigments ankaflavin (**163**), monascin (**164**), monasfluore A (**165**), and monasfluore B (**166**) and orange pigments monascorubrin (**167**) and rubropunctatin (**168**) (Figure S1). Western blot analysis revealed that the deletion of *Mrhos3* significantly elevated the acetylation level of H3K9, H4K12, and H3K18, as well as the total protein [77].

*U. virens* is the causal agent of rice false smut, which has recently become one of the most important rice diseases worldwide. Ustilaginoidins, sorbicillinoids, and ustiloxins are major mycotoxins produced by *U. virens* and greatly deteriorate grain quality and human health [97,98,99,100,101]. UvHos3 negatively regulates mycotoxin biosynthesis, particularly for ustilaginoidin and sorbicillinoid production, by modulating the acetylation level of H3K18 in *U. virens*. Unfortunately, the study did not reveal which mycotoxin was increased in this fungus [102].

### 3.3. Regulation of Class III HDACs on Biosynthesis of SMs in Fungi

Class III HDACs are sirtuin-type NAD^+^-dependent deacetylases; their activity is sensitive to intracellular NAD^+^ availability [103]. Class III HDACs that regulate fungal secondary metabolism include Hst2, Hst4, SirA, Sir2, SirD, and SirE (Table 2).

#### 3.3.1. Regulation of Hst2

Hst2 is also called HstB or SirT2. The deletion of *Fvhst2* in *F. verticillioides* led to an increased level of H4K16ac. Correspondingly, fumonisin B1 (FB1, **64**) production was increased, which meant that FB1 (**64**) biosynthesis was negatively impacted by *Fvhst2*. The ∆*Fvhst2* mutant increased vegetative growth, conidiation, and virulence when infecting sugarcane and maize [53].

Hst2 controlled the rate of mycelial growth in *U. virens*, retarded mycelial growth rates, and reduced viral pathogenicity, while it negatively regulated the biosynthesis of a variety of SMs. Unfortunately, the SMs were not identified [104].

#### 3.3.2. Regulation of Hst4

Hst4 is also called HstD or SirT4. AoHst4 is located upstream of LaeA, regulating fungal secondary metabolism and development in *A. oryzae*. When *AoHst4* is deleted, the production of kojic acid (**65**) increases [78].

The deletion of *HstD* in marine-derived *A. terreus* RA2905 led to the decreased production of lovastatin (**169**) and butyrolactones I (**91**) and II (**92**), as well as the increased production of terrein (**170**) (Figure S1). Furthermore, the loss of HstD in *A. terreus* resulted in a significant upregulation of H3K27 and H3K56 acetylation when compared to the wild-type strain, suggesting that the epigenetic functions of HstD, as a deacetylase, targeted H3K27 and H3K56 [79].

The disruption of *Mrhst4* in *M. ruber* significantly increased the yields of MonAzPs (*Monascus* azaphilone pigments), including ankaflavin (**163**), monascin (**164**), monasflore A (**165**), monasflore B (**166**), monascuburin (**167**), and rubropunctatin (**167**). The citrinin (**5**) content of ∆*Mrhst4* was also dramatically enhanced. RT-qPCR analysis showed that the absence of *Mrhst4* significantly increased the relative expression of citrinin (**5**) biosynthetic pathway genes, including *pksCT*, *mrl1*, *mrl2*, *mrl4*, *mrl6*, and *mrl7*. The Western blot assay suggested that the deletion of *Mrhst4* could significantly elevate the acetylation levels of H3K4, H3K9, H3K18, H3K56, and H4K12, but attenuated the lysine acetylation modification of H4Pan, H4K8, and H4K16 [80].

#### 3.3.3. Regulation of SirA

The genes involved in the biosynthesis of sterigmatocystin (**53**) and penicillin G (**54**) in *A. nidulans* were regulated by sirtuin A (SirA), which was associated with their promoters and repressed these structural genes via H4K16Ac deacetylation [81].

5-Methylmellein, which was a polyketide isolated from the culture broth of *Dihymobotryum rigidum* JCM 8837, inhibited the HDAC activity of SirA produced by *A. nidulans*. When 5-methylmellein was added to the medium at 100 μM, the production of sterigmatocystin (**53**) was increased 1.5-fold, which indicated that SirA negatively regulated the production of sterigmatocystin (**53**) [105]. Similarly, dihydrocoumarin (DHC, an inhibitor of the sirtuin family of HDACs) was used to treat *M. ruber* to increase production of three pigments monasfluol B (**171**), acetyl monasfluol B (**172**), and monascusazaphilone C (**173**) (Figure S1) while inhibiting the production of citrinin (**5**) [82].

The deletion of *sirA* in *A. nidulans* led to the increased production of sterigmatocystin (**53**) and austinol (**79**), which indicated that SirA was a negative transcriptional regulator of the secondary metabolism in *A. nidulans* [83].

#### 3.3.4. Regulation of Sir2

Sir2 is also called SirB. The deletion of *Fvsir2* in *F. verticillioides* led to the increased production of fumonisin B1 (**64**), which meant that FvHda1 negatively regulated fumonisin biosynthesis [53].

HDAC3, which was a member of the Sir2 family, catalyzed the deacetylation of lysine 56 of histone H3 (H3K56). Deleting *Hdac3* significantly reduced the tolerance of *M. robertsii* to oxidative stress from insects and plants, thereby decreasing the fungal ability to colonize the insect hemocoel and plant roots. HDAC3 achieved this by regulating the expression of three genes in the ergosterol (**121**) biosynthesis pathway, which included the lanosterol synthase gene *Las1*. The deletion of *Hdac3* or *Las1* reduced the ergosterol (**121**) content and impaired cell membrane integrity [84].

#### 3.3.5. Regulation of SirD

SirD is also called Sir4. The ∆*sirD* mutant of *Aspergillus kawachii* showed lower levels of acid-stable α-amylase activity and citric acid (**174**) (Figure S1) production. The SirD disruptant also showed a change in mycelial pigmentation and had higher sensitivity to cell wall biogenesis inhibitors such as calcofluor white and Congo red and reduced conidia formation. It indicated that SirD positively regulated secondary metabolism, cell wall integrity, and conidial development [85].

The overexpression of *Fvsir4* in *F. verticillioides* led to the decreased production of fumonisin B1 (**64**). Accordingly, the analysis of key FUM gene expression involved in FB1 toxin synthesis revealed significant decreases in FUM1, FUM8, and FUM19 in the *FvSirt4*-OE strain. This showed that FvHda1 negatively regulated the biosynthesis of fumonisin B1 (**64**) [53].

#### 3.3.6. Regulation of SirE

SirE (also called Sir5) is the abbreviation of sirtuin E. SirE is a NAD-dependent class III HDAC involved in global transcriptional regulation.

The deletion of *sirE* in *A. nidulans* led to decreased mycelial autolysis, conidiophore development, sterigmatocystin (**53**) biosynthesis, and the production of extracellular hydrolases. Moreover, the transcription of the genes involved in these processes was also decreased, indicating that SirE was an HDAC that up-regulated these activities [86].

The Δ*sirE* mutant increased aflatoxin B1 (**40**) production in *A. flavus*. Furthermore, the Δ*sirE* mutant displayed high sensitivity to osmotic stress and cell wall stress [87].

SirE was required for full virulence of *A. fumigatus*. In the Δ*sirE* strain, the biosynthesis of SMs was regulated negatively with the increased production of pseurotin A (**175**), brevinamide F (**176**), pyripyropene A (**177**), deacetyl pyriopyropene (**178**), and bis(methylthio)gliotoxin (**179**) (Figure S1) [88].

## 4. Conclusions

There are many regulation strategies to stimulate or inhibit the biosynthesis of fungal SMs, such as epigenetic regulation [13,106,107], transcriptional regulation [108,109], environmental signal regulation [110], and signal transduction regulation [111]. Among them, histone acetylation modification, a conservative post-translational epigenetic regulation, has been considered the most important strategy to regulate gene expression responsible for SM biosynthesis [112].

This review only summarized the regulation of histone acetylation in SM production in fungi. Actually, the acetylation of histone in fungi has many other biological functions, which are very important for fungi. For example, *GcnE* deficiency significantly reduced the radial growth of fungal colonies and number of conidiophores, as well as affected the formation of biofilm mucosa and increased gliotoxin (**6**) production in *A. fumigatus* [19]; *Hst2* deletion in *F. verticillioides* resulted in the increases of H4K16ac level and fumonisin B1 (**64**) production and also increased vegetative growth, conidiation, and virulence when infecting sugarcane and maize [53].

Many BGCs for SM biosynthesis in fungi are in a silent or low expression state under normal environmental conditions. Epigenetic regulation (i.e., histone acetylation modification) should be an effective strategy to activate the silent BGCs to produce new SMs by gene deletion/overexpression [112] or by using epigenetic modifiers [13,113], such as Hda1 modification, which has been proven to be a powerful way to activate silent gene clusters and has led to the biosynthesis of diverse SMs in *C. arbuscula* [65,66] and *P. fici* [73,74]. On the other hand, the BGCs for harmful SM production that are in an expressed state can also be inhibited to reduce their biosynthesis by a regulation strategy, such as HAT enzyme MrGcn5 positively regulating citrinin (**5**) biosynthesis in the fermentation process of *M. ruber* [28]. Furthermore, the cross-strategy of epigenetic regulation with global transcriptional regulation has been widely applied to various fungi, which has become one of the most important research directions in the biosynthesis of SMs in fungi [109,114,115].

In fungi, both HDACs and HATs can regulate many biological processes, such as SM biosynthesis, cell cycle progression, virulence, infection, stress response, DNA damage repair, and carbon source utilization [116]. It is worth noting that the lysine acetylation modification of histone has been considered the drug target for the creation of fungicides [1,117,118,119,120]. The impacts of histone acetylation modification on the biosynthesis of SMs in fungi have been confirmed and applied. However, their regulatory mechanisms are very complicated due to the functional complementarity of different HDACs and HATs. These specific regulatory mechanisms of HDACs and HATs are still largely unclear and require detailed investigation [47].

## Supplementary Materials

The following supporting information can be downloaded at: https://www.mdpi.com/article/10.3390/ijms26010025/s1.
