# Supplementary material for: Regulation of Histone Acetylation Modification on Biosynthesis of Secondary Metabolites in Fungi
Source: Int J Mol Sci. 2024 Dec 24;26(1):25. doi: 10.3390/ijms26010025 (PMC11720177; doi:10.3390/ijms26010025)
Supplement: Supplementary file 1 [file ijms-26-00025-s001.zip › ijms-3341541-supplementary-done.pdf]

International Journal of
Molecular Sciences

MDPI

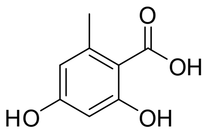

1. Orsellinic acid

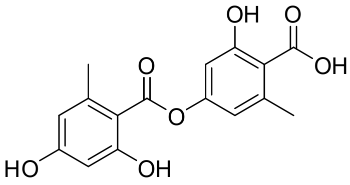

2. Lecanoric acid

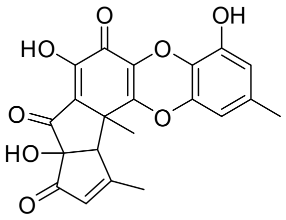

3. F-9775A

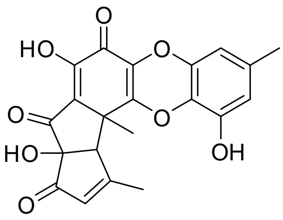

4. F-9775B

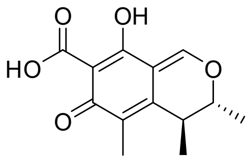

5. Citrinin

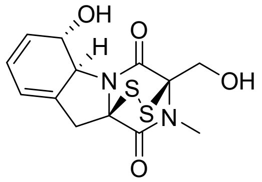

6. Gliotoxin

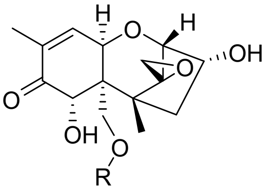

7. Deoxynivalenol, R=H
8. 15-Acetyldeoxynivalenol, R=$COCH_3$

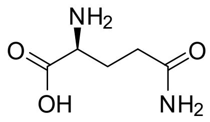

9. Glutamine

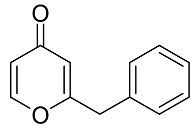

10. Nigerpyrone

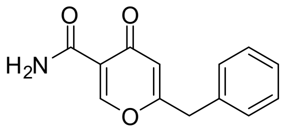

11. Carbonarone A

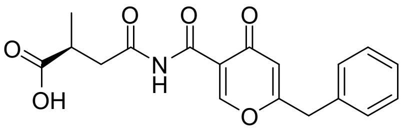

12. Pestalamide A

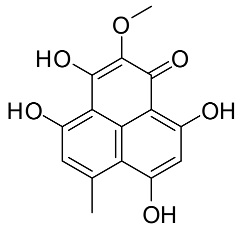

13. Funalenone

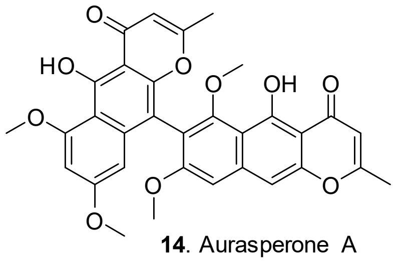

14. Aurasperone A

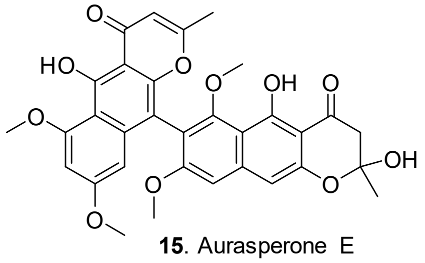

15. Aurasperone E

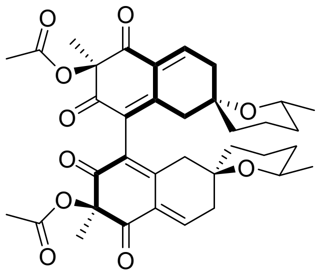

16. Cochliodone A

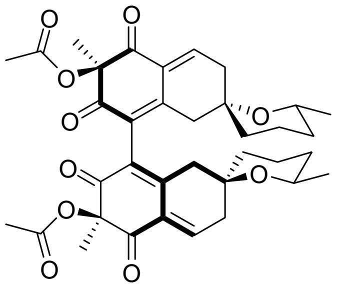

17. Cochliodone B

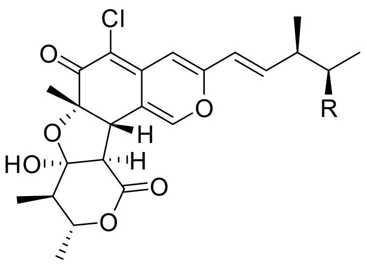

18. Chaetomugilin A, R=OH
19. Chaetomugilin D, R=H

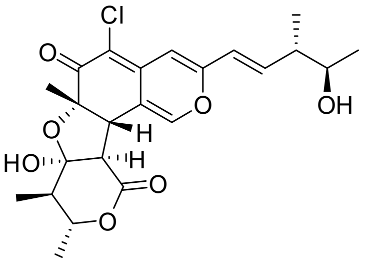

20. 11-*epi*-Chaetomugilin A

 https://doi.org/10.3390/xxxxx

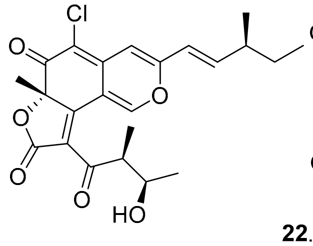

21. Chaetoviridin A

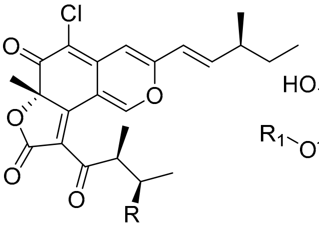

22. Chaetoviridin F, R=H
23. Chaetoviridin H, R=OH

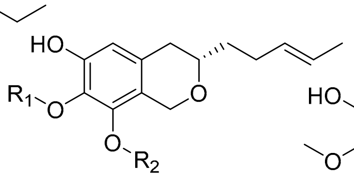

24. Meromuside A, R_1=Glu, R_2=H
25. Meromusdie B, R_1=H, R_2=Glu

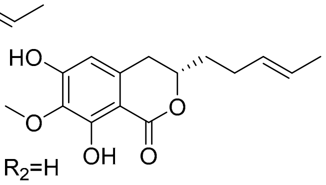

26. Meromuside C

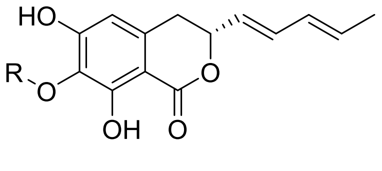

27. Meromuside D, R=Glu
28. Meromuside E, R=CH_3

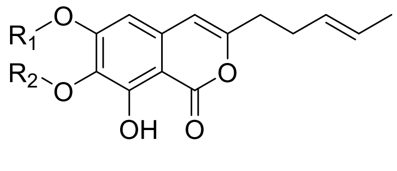

29. Meromuside F, R_1=H, R_2=CH_3
30. Meromuside G, R_1=CH_3, R_2=H
31. Meromuside H, R_1=H, R_2=Glu

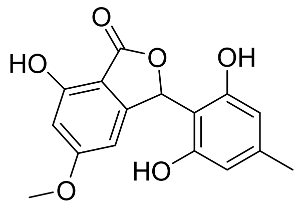

32. Meromuside I

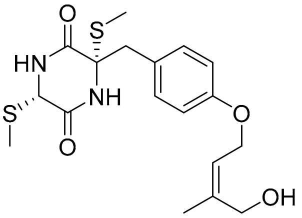

33. Meromutide A

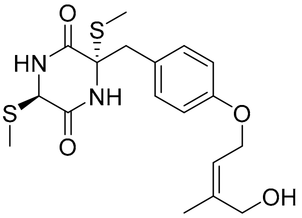

34. Meromutide B

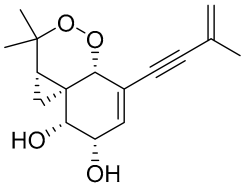

35. Monosporasol A

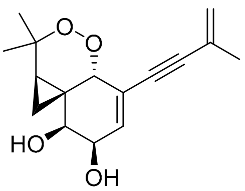

36. Monosporasol B

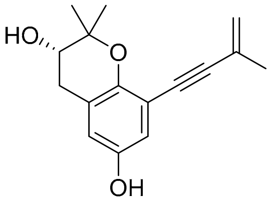

37. Pestaloficin C

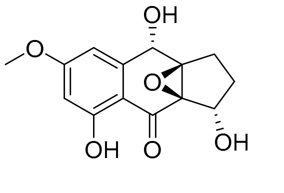

38. Arthrinone

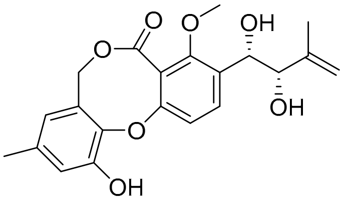

39. Pestalotiollide B

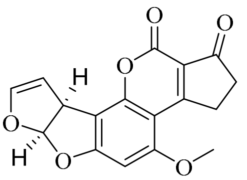

40. Aflatoxin B1

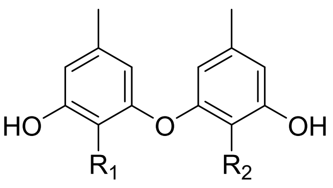

41. Diorcinol, R_1=H, R_2=H
42. Cordyol C, R_1=OH, R_2=H
43. Violaceol I, R_1=OH, R_2=OH

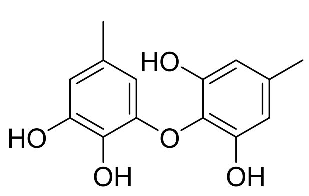

44. Violaceol II

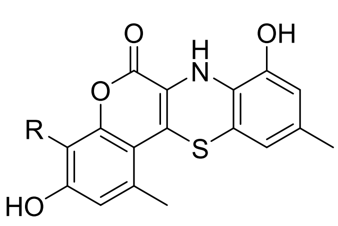

45. Pheofungin A, R=H
46. Pheofungin B, R=OH

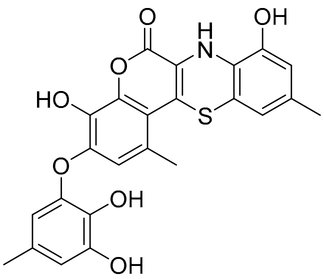
47. Pheofungin C

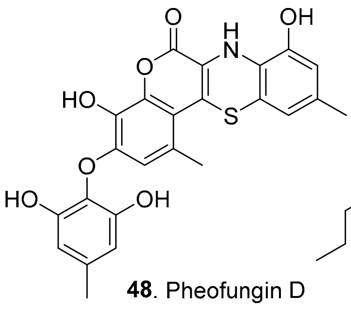
48. Pheofungin D

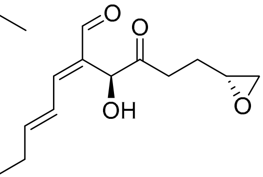
49. Mollipilin A

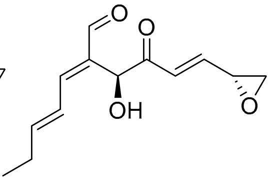
50. Mollipinin B

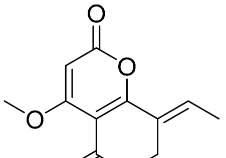
51. Coarctatin

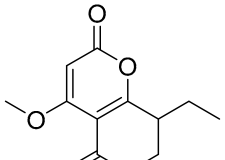
52. Dihydrocoarctatin

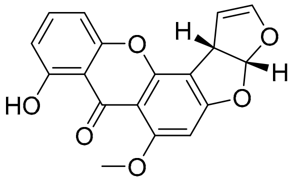
53. Sterigmatocystin

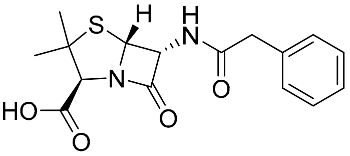
54. Penicillin G

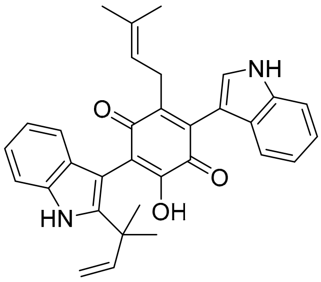
55. Terrequinone A

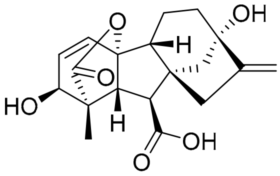
56. GA3

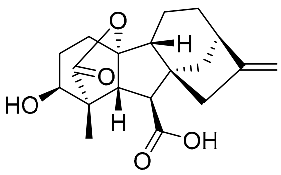
57. GA4

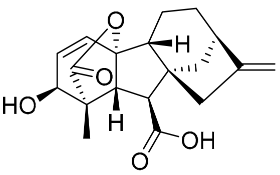
58. GA7

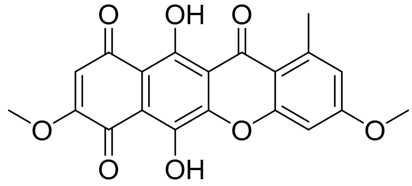
59. Bikaverin

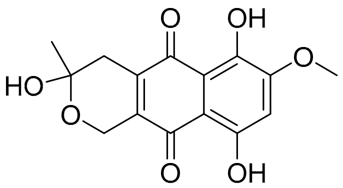
60. Fusarubin

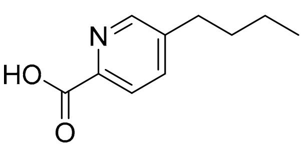
61. Fusaric acid

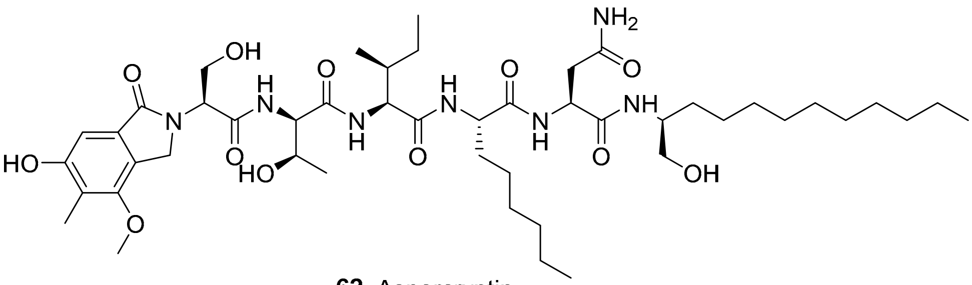
62. Aspercryptin

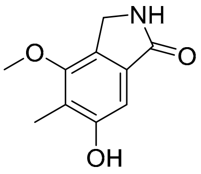
63. Cichorine

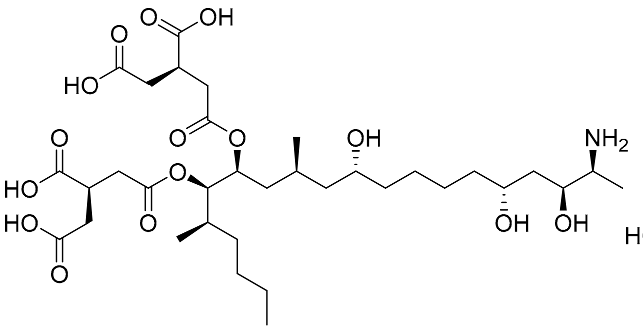

64. Fumonisin B1

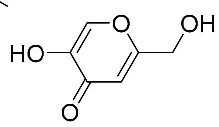

65. Kojic acid

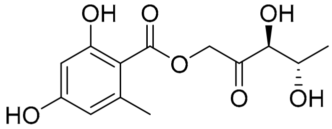

66. Globosumnone C

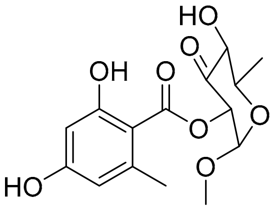

67. Orsellide A

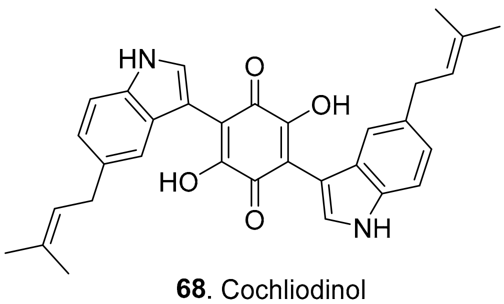

68. Cochliodinol

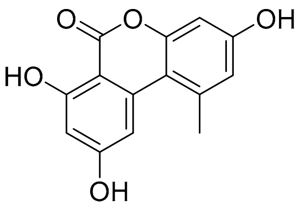

69. Alternariol

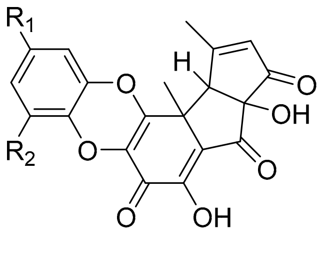

70. F 9775A, R_1=CH_3, R=OH
71. F 9775B, R_1=OH, R=CH_3

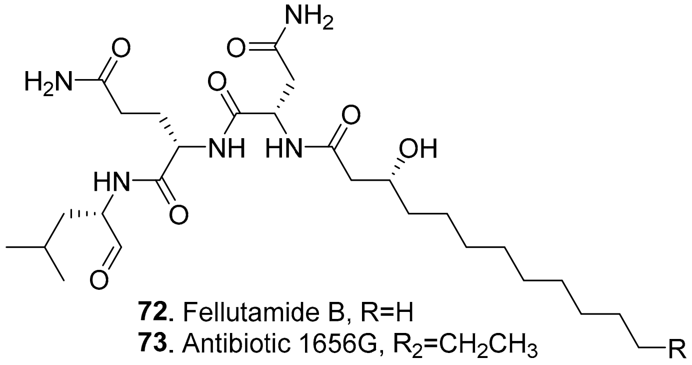

72. Fellutamide B, R=H
73. Antibiotic 1656G, R_2=CH_2CH_3

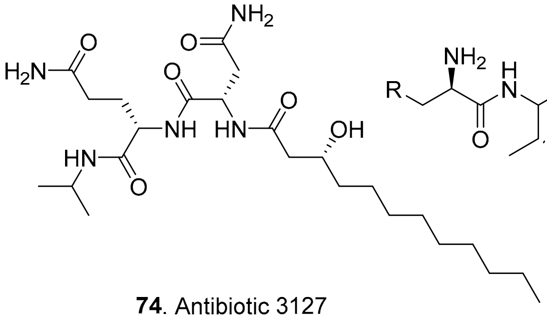

74. Antibiotic 3127

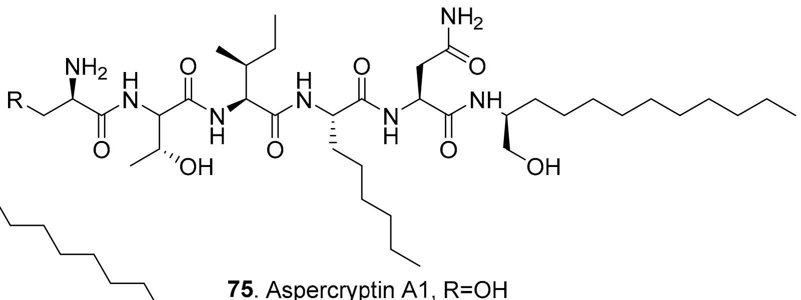

75. Aspercryptin A1, R=OH
76. Aspercryptin A2, R=H

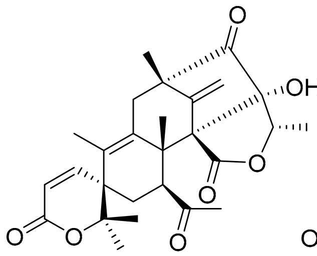

77. Isoaustinone

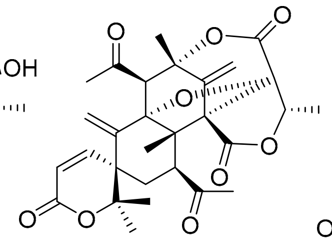

78. Acetoxydehydroaustin

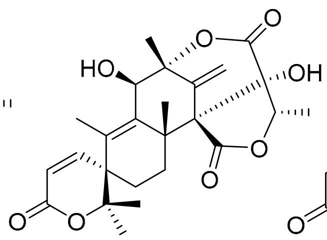

79. Austinol

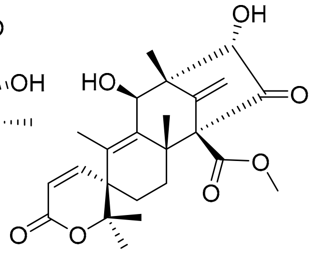

80. Austinoneol A

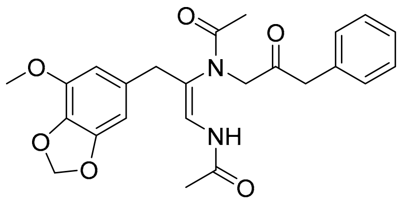

81. Brasiliamide A

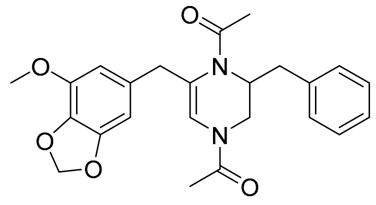

82. Brasiliamide B

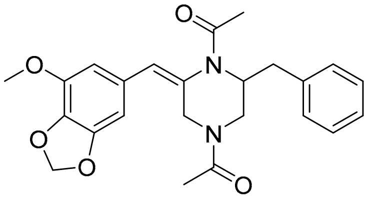

83. Brasiliamide C

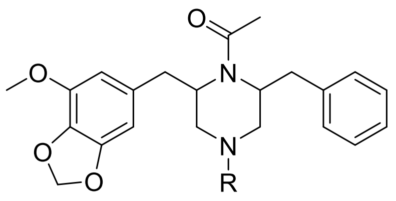

84. Brasiliamide D, R=$COCH_3$
85. Brasiliamide E, R=H

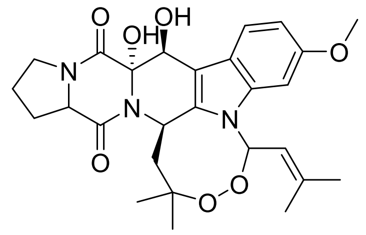

86. Verruculogen

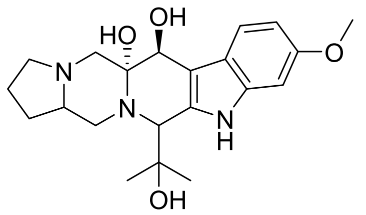

87. Verruculogen TR2

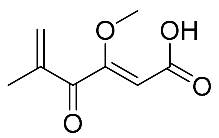

88. Penicillic acid

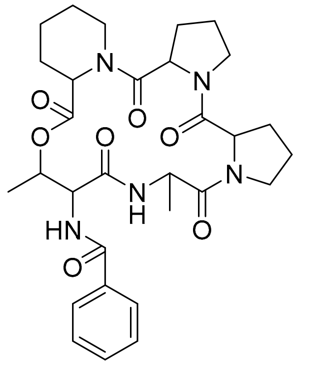

89. JBIR 114

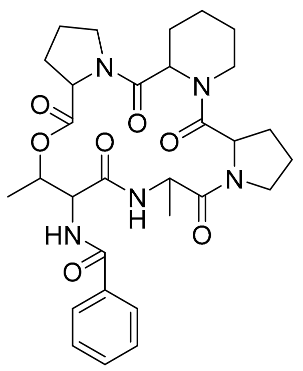

90. JBIR 115

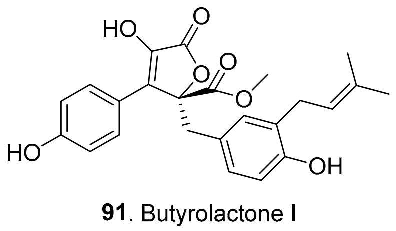

91. Butyrolactone **I**

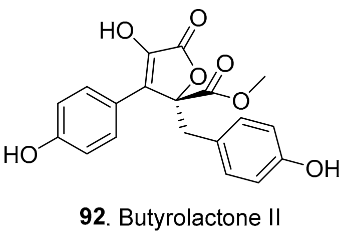

92. Butyrolactone II

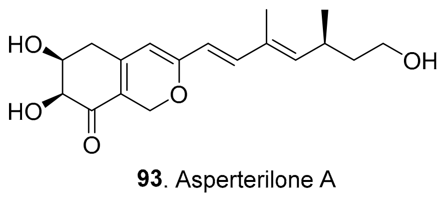

93. Asperterilone A

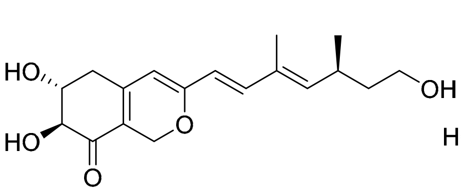

94. Asperterilone B

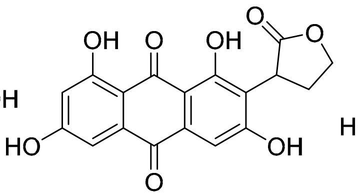

98. Paeciloquinone A

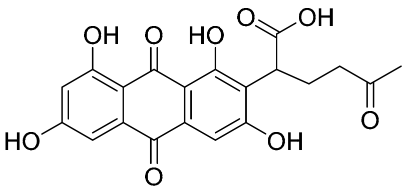

99. Paeciloquinone B

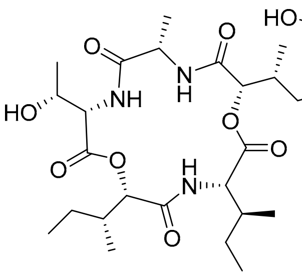

95. Arbumycin

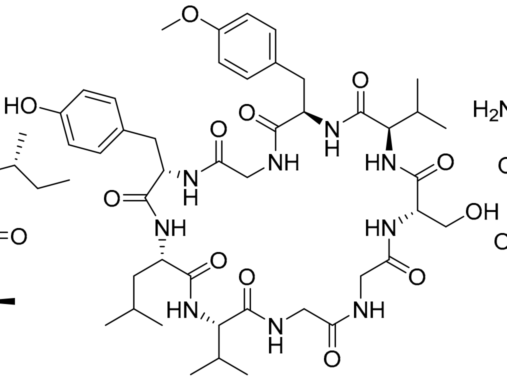

96. Arbumelin

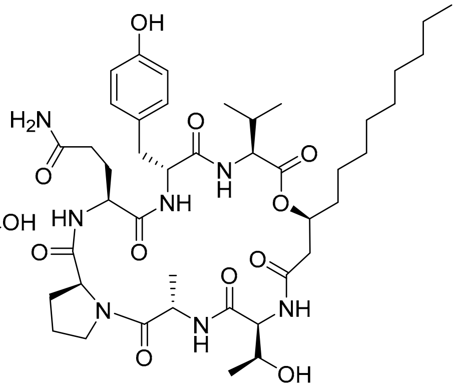

97. Verlamelin A

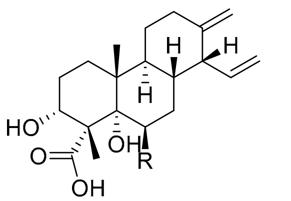

100. Zythiostromic acid A, R=OH
101. Zythiostromic acid B, R=H

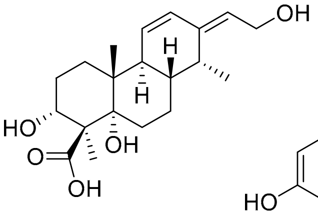

102. Arbusculic acid A

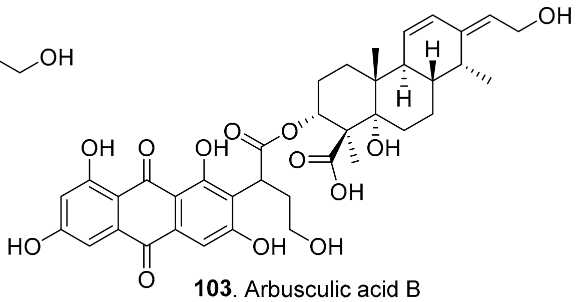

103. Arbusculic acid B

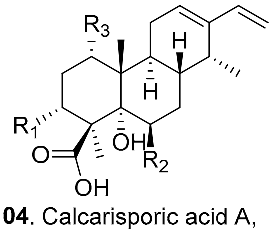

104. Calcarisporic acid A, R_1=OH, R_2= =O, R_3=H
105. Calcarisporic acid B, R_1=R_2=OH, R_3=H
106. Calcarisporic acid C, R_1=R_2=H, R_3=OH

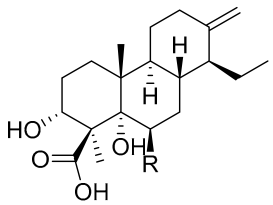

107. Calcarisporic acid D, R=OH
116. Hawaiinolide G, R= =O
117. 14-*epi*-Zythiostromic acid B, R=H

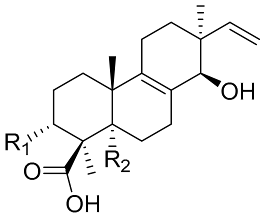

108. Calcarisporic acid E, R_1=R_2=OH
110. Calcarisporic acid G, R_1=H, R_2=OH
112. Calcarisporic acid I, R_1=OH, R_2=H

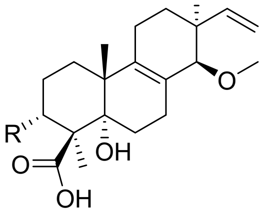

109. Calcarisporic acid F, R=OH
111. Calcarisporic acid H, R=H

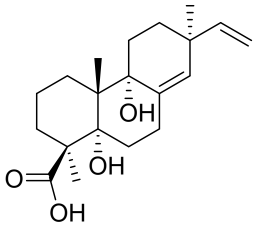

113. Calcarisporic acid J

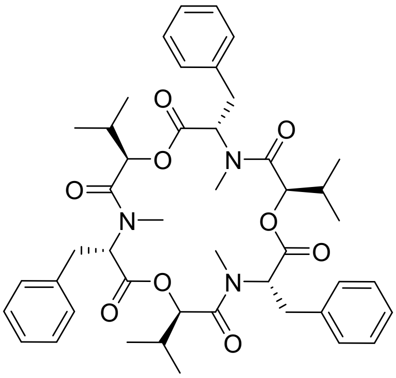

119. Beauvericin

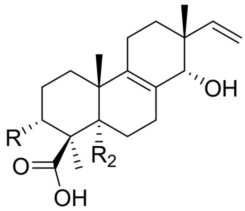

114. Calcarisporic acid K, R_1=OH, R_2=H
115. Calcarisporic acid L, R_1=H, R_2=OH

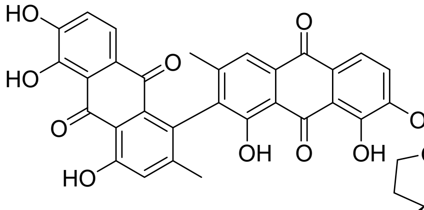

118. Cladofulvin

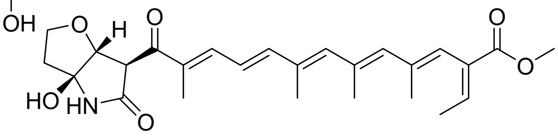

120. Fusarin A

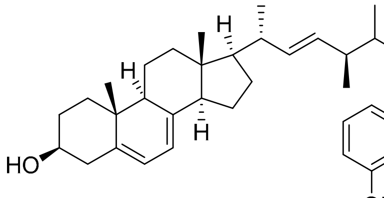

121. Ergosterol

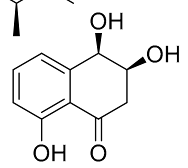

122. 3,4,8-Trihydroxytetralone

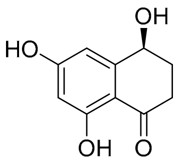

123. 4,6,8-Trihydroxytetralone

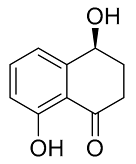

124. 4,8-Dihydroxytetralone

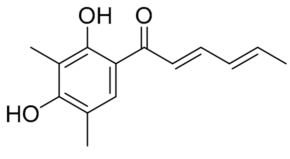

125. Sorbicillin

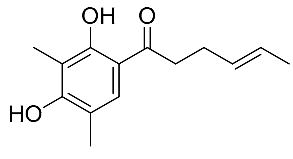

126. 2',3'-Dihydrosorbicillin

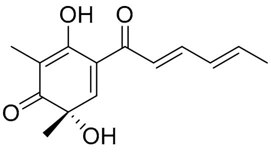

127. Sorbicillinol

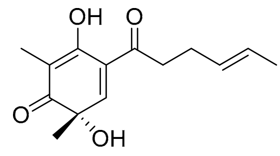

128. 2',3'-Dihydrosorbicillinol

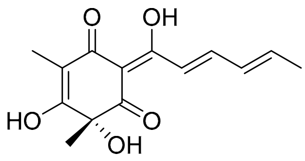

129. Oxosorbicillinol

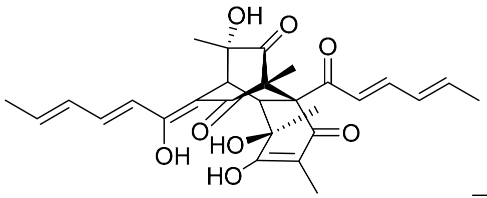

130. Bisorbicillinol

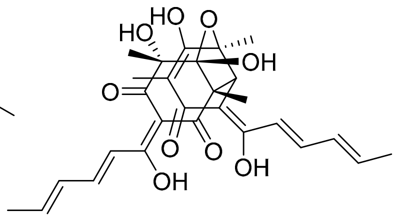

131. Bisvertinolone

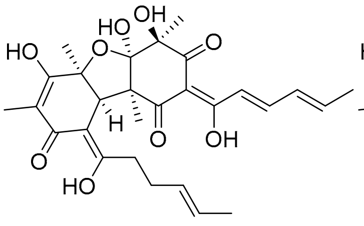

132. Dihydrobisvertinolone

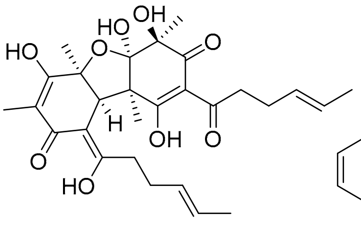

133. Tetrahydrobisvertinolone

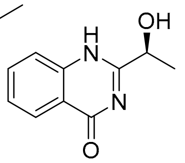

134. Chrysogine

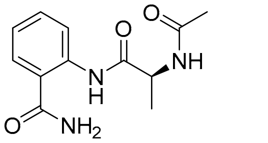

135. *N*-Acetylalanylanthranilamide

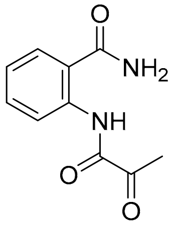

136. *N*-Pyrovoylanthranilamid

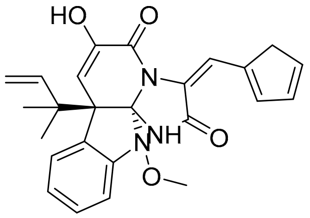

137. Meleagrin

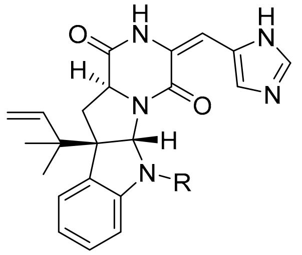

138. Roqueforine C, R=H
139. Roqueforine F, R=OCH_3

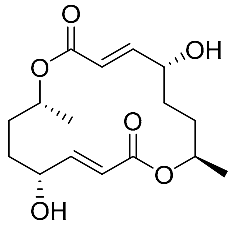

140. Ficiolide A

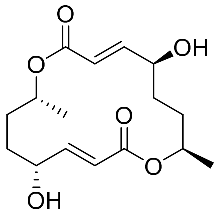

141. Ficiolide B

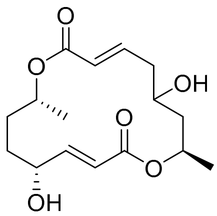

142. Ficiolide C

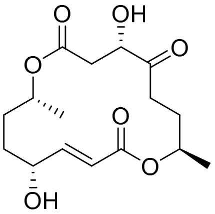

143. Ficiolide D

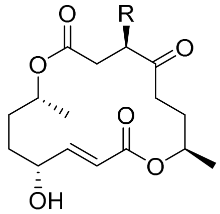

144. Ficiolide E, R=OH
145. Ficiolide F, R=H

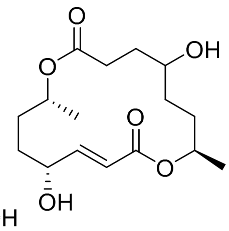

146. Ficiolide G

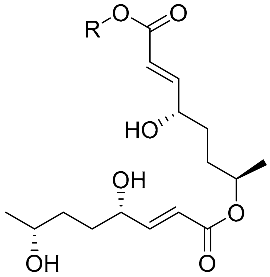

147. Ficiolide H, R=H
148. Ficiolide I, R=CH_3

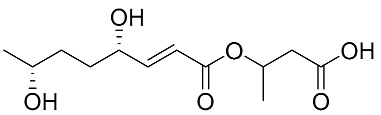

149. Ficiolide J

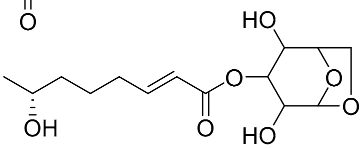

150. Ficiolide K

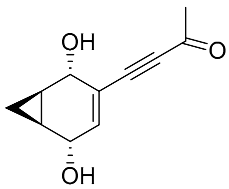

151. Pestaloficiol W

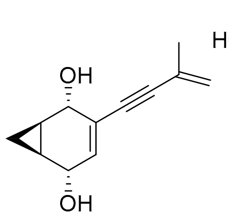

152. Asperpentyn

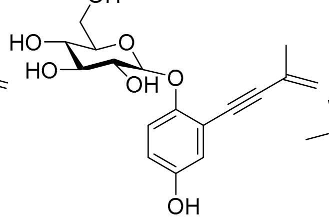

153. Pestaloficin X

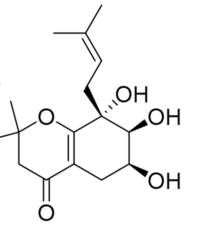

154. Pestaloficiol M

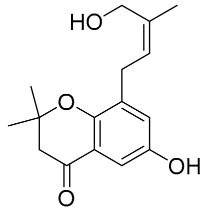

155. Festaloficin D

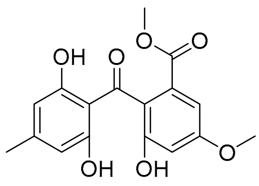

156. Isosulochrin

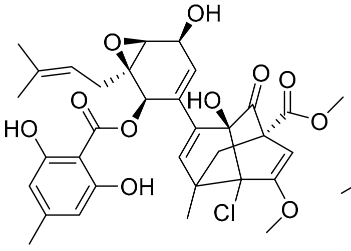

157. Chloropupukeananin

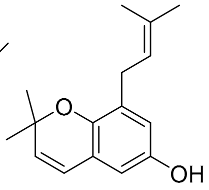

158. Pestalofiociol J

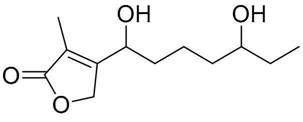

159. Hydroxyisoseiridin

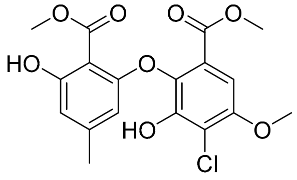

160. Pestheic acid

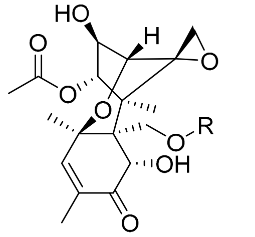

161. 4-Acetylnivalenol, R=H
162. 4,15-Diacetylnivalenol, R=$COCH_3$

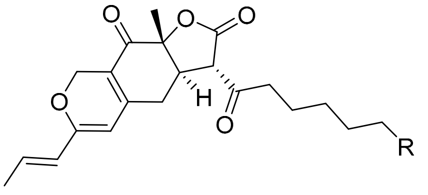

163. Ankaflavin, R=CH_2CH_3
164. Monascin, R=H

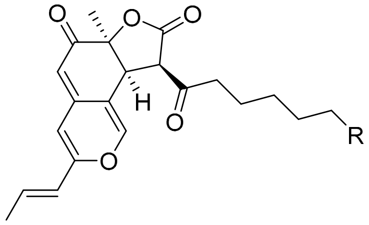

165. Monasfluore A, R=H
166. Monasfluore B, R=CH_2CH_3

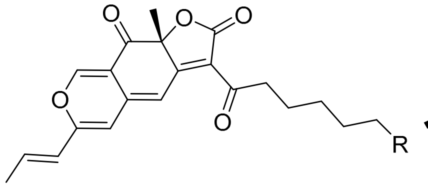

167. Monascorubrin, R=CH_2CH_3
168. Ruvropunctatin, R=H

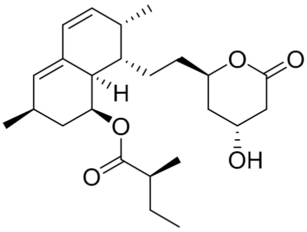

169. Lovastatin

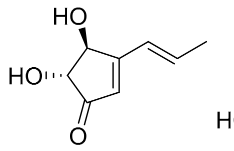

170. Terrein

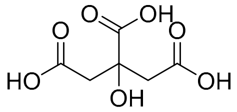

174. Citric acid

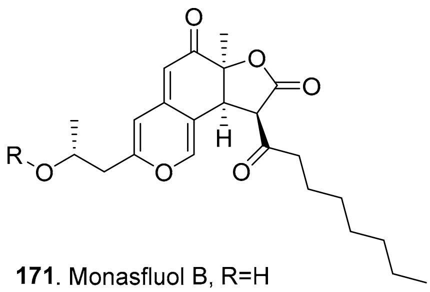

171. Monasfluol B, R=H
172. Acetyl monasfluol B, R=$COCH_3$

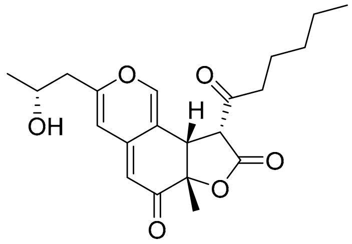

173. Monascusazaphilone C

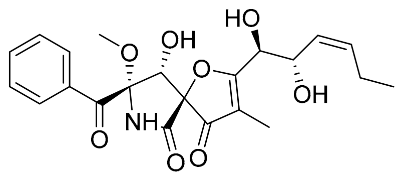

175. Pseurotin A

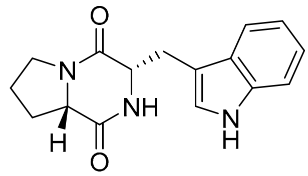

176. Brevianmide F

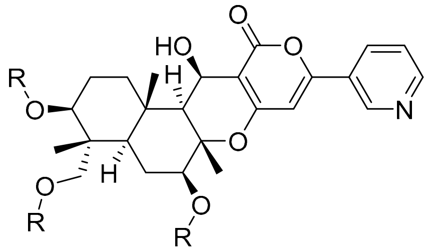

177. Pyripyropene A, R=$COCH_3$
178. Deacetyl pyriopyropene, R=H

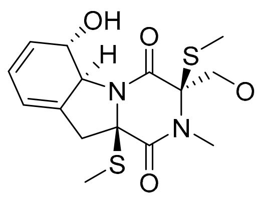

179. Bis(methylthio)gliotoxin

Figure S1. Structures of the compounds (**1**–**179**) produced by the fungi regulated by histone acetyltransferases (HATs) and histone deacetylases (HDACs).
